# Supplementary material for: Estimating individuals’ genetic and non-genetic effects underlying infectious disease transmission from temporal epidemic data
Source: PLoS Comput Biol. 2020 Dec 21;16(12):e1008447. doi: 10.1371/journal.pcbi.1008447 (PMC7785229; doi:10.1371/journal.pcbi.1008447)
Supplement: S1 Appendix — (PDF) [file pcbi.1008447.s001.pdf]

S1 Appendix: Recovery dynamics

Fig S1 shows examples of gamma distributions for three different values of shape parameter *k=1, 2* and *10*. A common approach when modelling epidemics is to assume *k=1*, corresponding to the Markovian assumption of constant recovery probability. From a biological point of view, however, this is rather unrealistic. Typically after infection, the host's immune system takes some time to respond, *e.g.* to generate the appropriate antibodies to fight off the infection. Thus, for many real diseases *k* may be large.

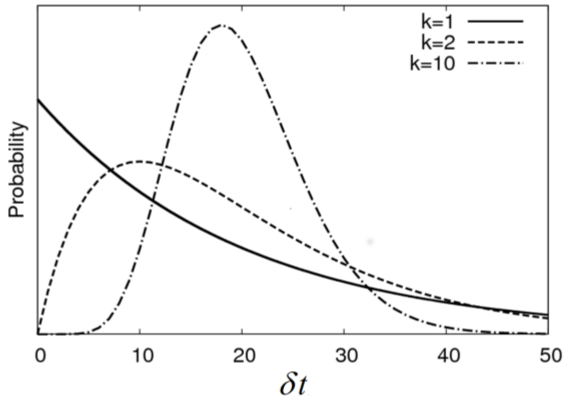

Fig S1. Infection duration. Shows the probability distribution for an individual to recover from their infection a time δt after they were infected.

Consequently, incorporation of a gamma distributed infection duration (which is characterised by two parameters, a mean and a shape parameter, instead of just one for the exponential distribution) allows the model to more realistically capture true disease dynamics.
