# Supplementary material for: Estimating individuals’ genetic and non-genetic effects underlying infectious disease transmission from temporal epidemic data
Source: PLoS Comput Biol. 2020 Dec 21;16(12):e1008447. doi: 10.1371/journal.pcbi.1008447 (PMC7785229; doi:10.1371/journal.pcbi.1008447)
Supplement: S2 Appendix — (PDF) [file pcbi.1008447.s002.pdf]

S2 Appendix: Derivation of the likelihood

The likelihood in Eq.(6) represents the probability the model in section 2.1 generates a certain set of events ξ, assuming a given set of parameters θ. This can be calculated by multiplying the probabilities for each of the individual sampling steps used in the simulation procedure, as described in S6 Appendix.

During initialisation of a given contact group, the infection duration for the initially infected individual *j* is sampled with probability

$$F_\Gamma(\delta t_j \mid w_j, k). \quad \text{(A1)}$$

We now consider each event *e* in turn. In step 1 of S6 Appendix, the inter-event time Δt is sampled from the exponential distribution $\Lambda_e e^{-\Lambda_e \Delta t}$ (where $\Lambda_e(t_e)$ is the total infection rate Eq.(7) evaluated immediately prior to t_e, the time of event *e*). Two possibilities exist for event *e*:

1. **It is a recovery event**. Considering the algorithm in S6 Appendix, step 2(a) would have been branched to, and this only happens if the sampled value for Δt is greater than the observed inter-event time t_e-t_{e-1}. The probability of this is calculated from the following integral:

$$\int_{t_e - t_{e-1}}^{\infty} \Lambda_e e^{-\Lambda_e(t_e)\times\Delta t} d\Delta t = e^{-\Lambda_e(t_e)\times(t_e - t_{e-1})}. \quad \text{(A2)}$$

2. **It is an infection event**. Again, considering the algorithm in S6 Appendix, step 2(b) would have been branched to, and this happens with probability $\Lambda_e e^{-\Lambda_e \times (t_e - t_{e-1})}$ (which comes from the exponential distribution above). The individual *j* which becomes infected is selected with probability λ_j / Λ_e. Finally, the infection duration of that individual δt_j is sampled from a gamma distribution with probability density function $F_\Gamma(\delta t_j \mid w_j, k)$. Combining these three contributions gives an overall probability:

$$\lambda_j e^{-\Lambda_e(t_e)\times(t_e - t_{e-1})} F_\Gamma(\delta t_j \mid w_j, k). \quad \text{(A3)}$$

Multiplying the results from Eqs.(A1), (A2) and (A3) for all the infection and recovery events leads to the likelihood for a single contact group of:

$$L(\xi \mid \theta) = \left(\prod\nolimits_j \lambda_j\right)\left(\prod\nolimits_e e^{-\Lambda_e(t_e)\times(t_e - t_{e-1})}\right)\left(\prod\nolimits_m F_\Gamma(\delta t_m \mid w_m, k)\right), \quad \text{(A4)}$$

where *j* goes over individuals that become infected (*excluding* those which initiate epidemics), *m* also goes over individuals that become infected but *including* those which initiate epidemics and *e* goes over both infection and recovery events (with corresponding event times t_e).

Since contact groups are assumed to be independent, the likelihood for multiple contact groups is simply the product of each separate one, as shown in Eq.(6).
