# Supplementary material for: Estimating individuals’ genetic and non-genetic effects underlying infectious disease transmission from temporal epidemic data
Source: PLoS Comput Biol. 2020 Dec 21;16(12):e1008447. doi: 10.1371/journal.pcbi.1008447 (PMC7785229; doi:10.1371/journal.pcbi.1008447)
Supplement: S3 Appendix — (PDF) [file pcbi.1008447.s003.pdf]

S3 Appendix: Prior definitions for model parameters

The default prior in SIRE (which can be altered), which is used for all the results in this paper, is largely uninformative but does place upper and lower bounds on many of the key parameters to stop them straying into biologically unrealistic regimes. Bounding parameters in this way was found to be especially important when considering relatively uninformative data scenarios when unbounded flat priors could lead to improper posterior probability distributions. Specifically, a uniform prior between -2.3 and 2.3 was chosen for a_g. This corresponds to assuming that it is biologically unrealistic for a single SNP to change the susceptibility of individuals by more than a factor of 100 between *AA* and *BB* individuals[1]. An identical uniform prior was also placed on a_f, a_r and on each of the fixed effects in $\boldsymbol{b}_g$, $\boldsymbol{b}_f$ and $\boldsymbol{b}_r$. Similarly, a uniform prior between -3.45 and 3.45 was placed on each of the residuals $\boldsymbol{\varepsilon}_g$, $\boldsymbol{\varepsilon}_f$ and $\boldsymbol{\varepsilon}_r$, This larger range reflects a potential factor of 1000 variation across individuals (chosen to be larger as residual contributions account for all other SNPs as well as non-genetic factors, as opposed to just the effect of the single SNP under analysis).

The scaled dominance factors Δ_g, Δ_f, Δ_r, were chosen to have uniform priors between 1 and -1, *i.e.* going from complete dominance of *A* to complete dominance of *B* [2]. The prior for the shape parameter *k* was chosen to be uniform between 1 and 10, where 1 represents a Poisson random process and 10 represents a situation in which recovery times of individuals are highly concentrated around their mean.

Because parameters β and γ depend on the timescale over which the epidemic is measured[3], which is partly pathogen specific, placing informative priors on β and γ by default is not appropriate (although it can be done). Instead a uniform prior between 0 and 20 was placed on the equivalent basic reproductive ratio R_0 [4], which *is* a dimensionless quantity.

---

[1] This factor comes from the exponential dependency in Eq.(1) coupled with the result $e^{2\times 2.3}$ =100.

[2] If over dominance is considered a possibility, this prior distribution would be extended.

[3] E.*g.* if measurements are made in hours then β and γ would be very different to if they were made in days.

[4] Defined by $R_0=\beta(\langle N\rangle-1)/\gamma$, where ⟨N⟩ is the average population size of contact groups as taken from the data, this represents the number of infections one typically infectious individual generates on average over the course of its infectious period in an otherwise uninfected population.
