# Supplementary material for: Estimating individuals’ genetic and non-genetic effects underlying infectious disease transmission from temporal epidemic data
Source: PLoS Comput Biol. 2020 Dec 21;16(12):e1008447. doi: 10.1371/journal.pcbi.1008447 (PMC7785229; doi:10.1371/journal.pcbi.1008447)
Supplement: S4 Appendix — (PDF) [file pcbi.1008447.s004.pdf]

S4 Appendix: MCMC procedure

Markov chain Monte Carlo (MCMC) produces a list of (correlated) parameter θ^q and event time ξ^q samples drawn from the posterior probability distribution in Eq.(5), where q indexes sample number.

1) Initialisation – MCMC is initially started with some set of parameters θ^1 and event times ξ^1 consistent with the data y. In the case of parameters, θ^1 is simply sampled from the prior distribution $\pi(\theta)$. For events, initialisation depends on the available data: DS1) events are defined by the data, DS2) infection times are exponential sampled backwards in time from the first observed recovery time[1], DS3) recovery times are exponentially sampled forwards in time from the final observed infection time[2], DS4) infection and recovery times are sampled uniformly in the time intervals identified between successive diagnostic tests, and DS5) events within the observation period are defined by the data, infection events prior to this time period are exponentially sampled backward in time and similarly recoveries events after this time period are exponentially sampled forward in time.

2) **Iteration** - MCMC operates by proposing changes to the model parameters θ and event times ξ and accepting or rejecting these changes in accordance with a Metropolis-Hastings probability. In this way the underlying dynamics are able to explore all potential possibilities consistent with the observations.

A single MCMC "update" consists of making the following sets of proposals:

MCMC UPDATE

Parameters – Each individual parameter in θ=(β, γ, k, a_g, a_f, a_r, Δ_g, Δ_f, Δ_r, $\boldsymbol{b_g}$, $\boldsymbol{b_f}$, $\boldsymbol{b_r}$, $\boldsymbol{\varepsilon_g}$, $\boldsymbol{\varepsilon_f}$, $\boldsymbol{\varepsilon_r}$, $\boldsymbol{\Sigma}$, $\boldsymbol{G}$, σ_G), denoted by θ_j, is considered in turn. A proposed value is drawn from a normal distribution centred on the parameter's current chain value

$$\theta_j^p \sim Norm(\theta_j^q, J_j^2), \tag{A1}$$

with all other parameters in θ^p remaining the same as in θ^q (if this produces an inconsistent value, *e.g.* β becomes negative, the proposal is immediately rejected). The proposal is accepted with Metropolis-Hastings probability

$$P_{MH} = \min\left(1, \frac{L(\xi^p|\theta^p)\pi(\theta^p)}{L(\xi^q|\theta^q)\pi(\theta^q)}\right). \tag{A2}$$

If accepted, we set θ^{q+1}= θ^p else θ^{q+1}= θ^q with ξ^{q+1}= ξ^q.

Tuning $J_{j(A3)}$ in Eq.(A1) is important. If it is too large, very few proposals will be accepted and if too small, mixing will be slow. Motivated by adaptive MCMC [2, 3], a robust heuristic method for optimising J_j within the burn-in period is as follows. Initially, J_j is set to a small quantity. Each time a proposed change on parameter j is accepted J_j is updated according to

$$J_j^{new} = J_j \times 1.01, \tag{A4}$$

and when rejected

[1] The standard deviation in recovery times is used to set the decay rate.
[2] The standard deviation in infection times is used to set the decay rate.

$$J_j^{new} = J_j \times 0.995. \quad \text{(A5)}$$

These numerical factors are chosen for two reasons: Firstly, the updates in Eqs.(A4) and (A5) balance each other out when acceptance occurs around 33% of the time, leading to a steady state solution for J_j. Secondly, they are chosen to be sufficiently close to *1* to prevent large fluctuations in J_j, but sufficiently fa6r to allow the steady state solution to be found within the burn-in period.

In addition to the single parameter proposals outlined above, joint proposals are also made on residuals along with their corresponding covariance matrix, as described in S5 Appendix. The reason these joint updates are necessary is that $\boldsymbol{\Sigma}$ and $\boldsymbol{\varepsilon}$ are often highly correlated within the model (as determined by Eq.(9)). In many cases the data provides little information regarding $\boldsymbol{\Sigma}$ itself, so these correlations can lead to extremely slow mixing.

Events – In situations in which ξ is not precisely known (*i.e.* data scenarios other than DS1), the unknown latent event times must be stochastically changed in accordance with (to enable sampling from) the posterior distribution. Each individual *j* is considered in turn. In the case of proposing changes to the infection time of *j*, the following normal distribution is sampled from:

$$t_j^{\mathrm{I},p} \sim Norm(t_j^{\mathrm{I},q}, J_{I,j}^2), \quad \text{(A6)}$$

with all other event times in $\xi^p=(t^{\mathrm{I}},t^{\mathrm{R}})$ remaining the same as in ξ^q. If this proposed infection time exceeds the recovery time $t_j^{R,q}$ the proposal is immediately rejected (infections cannot occur after recoveries). The proposal in Eq.(A6) is accepted with probability

$$P_{MH} = \min\left(1, \frac{\pi\left(y|\xi^p\right)L(\xi^p|\theta^p)}{\pi\left(y|\xi^q\right)L(\xi^q|\theta^q)}\right). \quad \text{(A7)}$$

If accepted, we set $\xi^{q+1}= \xi^p$ else $\xi^{q+1}= \xi^q$ with $\theta^{q+1}= \theta^q$.

A similar proposal to Eq.(A6) is used to change recovery times. The jumping parameters $J_{I,j}$ and $J_{R,j}$ are again tuned to give an average acceptance probability of around 33% (using the same method as in Eqs.(A4) and (A5) above). Furthermore, there are data scenarios in which it is not possible to tell whether an individual has become infected or not. In these cases it is necessary to include additional proposals which insert infection/recovery event pairs and, conversely, other proposals which remove them.

Each of the proposals above have been optimised to calculate only those parts of the likelihood that change, *e.g.* if the infection time of individual *j* is altered then only the change in likelihood in the period between then initial and proposed times within *j*'s contact group needs to be calculated. Furthermore, when making some sets of proposals computational speed is substantially increased by pre-calculating terms in the likelihood which remain unchanged.

References

1. Pooley C, Bishop S, Doeschl-Wilson A, Marion G. Posterior-based proposals for speeding up Markov chain Monte Carlo. *Royal Society open science* 2019;*6*:190619.
2. Andrieu C, Thoms J. A tutorial on adaptive MCMC. Stat Comput. 2008;18:343-73.
3. Roberts GO, Rosenthal JS. Examples of Adaptive MCMC. J Comput Graph Stat. 2009;18:349-67.
