# Supplementary material for: Estimating individuals’ genetic and non-genetic effects underlying infectious disease transmission from temporal epidemic data
Source: PLoS Comput Biol. 2020 Dec 21;16(12):e1008447. doi: 10.1371/journal.pcbi.1008447 (PMC7785229; doi:10.1371/journal.pcbi.1008447)
Supplement: S5 Appendix — (PDF) [file pcbi.1008447.s005.pdf]

S5 Appendix: Posterior-based proposals for residuals

This appendix describes special joint proposals in the residuals ($\mathbf{\Sigma},\boldsymbol{\varepsilon}$) which are used to aid MCMC mixing. Specifically three type of proposal are considered: 1) those which randomly make changes to Σ_{gg}, Σ_{gf}, or Σ_{gr} and stochastically alter $\boldsymbol{\varepsilon}_g$ (with everything else kept fixed), 2) those which randomly make changes to Σ_{ff}, Σ_{gf}, or Σ_{rr} and stochastically alter $\boldsymbol{\varepsilon}_f$ (with everything else kept fixed), and 3) those which randomly makes changes to Σ_{rr}, Σ_{gr}, or Σ_{fr} and stochastically alter $\boldsymbol{\varepsilon}_r$ (with everything else kept fixed). Here we describe just one of these possibilities, but others can be found by suitably permuting indices.

So-called "posterior-based proposals" (PBPs) [1] consist of three steps:

Step 1 – A proposed is made to one of the parameters in the model. In this example

$$\Sigma_{gg}^{p} \sim Norm(\Sigma_{gg}^{q}, J_{\Sigma_{gg}}^{2}), \tag{A1}$$

with the value of $J_{\Sigma_{gg}}$ tuned to give an average acceptance probability of around 33% (using the same method as in S3 Appendix).

Step 2 – Each element $\varepsilon_{g,j}$ in the $\boldsymbol{\varepsilon}_g$ is considered in turn. The values of means $\mu_{q,j}$ and $\mu_{p,j}$ and standard deviations $\sigma_{q,j}$ and $\sigma_{p,j}$ are calculated such that the normal distributions they characterise approximate, to some level of accuracy, the true posterior distributions for $\varepsilon_{g,j}$ in the initial and proposed states (details on how this is done are discussed below). If $\sigma_{p,j}>\sigma_{q,j}$ then for each individual *j* the proposed residuals are sampled using

$$\varepsilon_{g,j}^{p} \sim Norm\left(\mu_{p,j} + \sqrt{\kappa + (1-\kappa)\frac{\sigma_{p,j}^{2}}{\sigma_{q,j}^{2}}}(\varepsilon_{g,j}^{q} - \mu_{q,j}), \kappa(\sigma_{p,j}^{2} - \sigma_{q,j}^{2})\right), \tag{A2}$$

where κ is a tuneable constant set to 0.03, and when $\sigma_p \leq \sigma_q$

$$\varepsilon_{g,j}^{p} \sim Norm\left(\mu_{p,j} + \sqrt{\kappa + (1-\kappa)\frac{\sigma_{q,j}^{2}}{\sigma_{p,j}^{2}}}\frac{\sigma_{p,j}^{2}}{\sigma_{q,j}^{2}}(\varepsilon_{g,j}^{q} - \mu_{q,j}), \kappa\frac{\sigma_{p,j}^{2}}{\sigma_{q,j}^{2}}(\sigma_{q,j}^{2} - \sigma_{p,j}^{2})\right). \tag{A3}$$

The somewhat complicated expressions in Eqs.(A2) and (A3) are specifically designed such that if the posterior *is* truly represented by the normal approximations (as characterised by $\mu_{q,j}$, $\mu_{p,j}$, $\sigma_{q,j}$ and $\sigma_{p,j}$), the proposal is accepted with probability 1.

Step 3 – The proposed combination $\Sigma_{gg}^{p}, \boldsymbol{\varepsilon}_{g}^{p}$ is accepted or rejected with Metropolis-Hastings probability

$$P_{MH} = \min\left\{1, \frac{\pi(y|\xi^{p})\pi(\xi^{p}|\theta^{p})\pi(\theta^{p})}{\pi(y|\xi^{q})\pi(\xi^{q}|\theta^{q})\pi(\theta^{q})}\prod_{j}\frac{f_{norm}(\varepsilon_{g,j}^{q}|\mu_{q,j},\sigma_{q,j})}{f_{norm}(\varepsilon_{g,j}^{p}|\mu_{p,j},\sigma_{p,j})}\right\}, \tag{A4}$$

where *j* goes over all individuals and

$$f_{\text{norm}}(x \mid \mu, \sigma) = \frac{1}{\sqrt{2\pi\sigma^{2}}} e^{-\frac{(x-\mu)^{2}}{2\sigma^{2}}} \tag{A5}$$

is the Gaussian probability density function.

Optimisation

For efficient implementation of this approach it is necessary to generate $\mu_{q,j}$, $\mu_{p,j}$, $\sigma_{q,j}$ and $\sigma_{p,j}$ in a suitable manner. One approximation is to set them to values implied by prior in Eq.(9):

$$\begin{aligned} \mu_{q,j} &= -(M^q_{gf}\varepsilon_{f,j} + M^q_{gr}\varepsilon_{r,j})/M^q_{gg}, \qquad \sigma_{q,j} = 1/M^q_{gg}, \\ \mu_{\mathrm{p},j} &= -(M^p_{gf}\varepsilon_{f,j} + M^p_{gr}\varepsilon_{r,j})/M^p_{gg}, \qquad \sigma_{p,j} = 1/M^p_{gg}, \end{aligned} \qquad \text{(A6)}$$

where **M** is the inverse of the covariance matrix $\mathbf{\Sigma}^{-1}$. This approximation leads to so-called "model-based proposals" (MBP). A more accurate approximation (because it takes into account the observed events, which themselves are informed by the data) is given by

$$\begin{aligned} \mu_q &= -(M^q_{gf}\varepsilon_{f,j} + M^q_{gr}\varepsilon_{r,j})/M^q_{gg} + \left.\frac{\partial \log\left(L(\xi^q \mid \theta^q)\right)}{\partial \varepsilon^q_{g,j}}\right|_{\varepsilon^q_{g,j}=0} \sigma^2_{q,j}, \qquad \sigma_{q,j} = 1/M^q_{gg}, \\ \mu_p &= -(M^p_{gf}\varepsilon_{f,j} + M^p_{gr}\varepsilon_{r,j})/M^p_{gg} + \left.\frac{\partial \log\left(L(\xi^p \mid \theta^p)\right)}{\partial \varepsilon^p_{g,j}}\right|_{\varepsilon^p_{g,j}=0} \sigma^2_{p,j}, \qquad \sigma_{p,j} = 1/M^p_{gg},, \end{aligned} \qquad \text{(A7)}$$

and this gives a first order "posterior-based proposal" (PBP). Although Eq.(A7) is computationally slower to calculate than Eq.(A6) (because it contains gradients in the log-likelihood), it can lead to larger jumps in parameter space compared to a corresponding MBP resulting in improved mixing. Empirically it was found that applying PBPs to parameters relating to susceptibility and recoverability and MBPs to parameters relating to infectivity was the fastest approach to take.

References

1. Pooley C, Bishop S, Doeschl-Wilson A, Marion G. Posterior-based proposals for speeding up Markov chain Monte Carlo. *Royal Society open science* 2019;*6*:190619.
