# Supplementary material for: Estimating individuals’ genetic and non-genetic effects underlying infectious disease transmission from temporal epidemic data
Source: PLoS Comput Biol. 2020 Dec 21;16(12):e1008447. doi: 10.1371/journal.pcbi.1008447 (PMC7785229; doi:10.1371/journal.pcbi.1008447)
Supplement: S6 Appendix — (PDF) [file pcbi.1008447.s006.pdf]

S6 Appendix: Simulation

The Doob-Gillespie algorithm [1] provides a means of taking into account inherent stochasticity in Markovian compartmental models (*i.e.* models for which the transition rates depend solely on the current state of the system). The model used in this paper combines Markovian infection transitions with more realistic non-Markovian recovery dynamics. Below we describe how these recovery events are incorporated into the standard Doob-Gillespie framework.

The purpose of this procedure is to build up a time ordered sequence of infection and recovery event times indexed by event number *e*. The following notation is used: t_e is the event time, x_e is the event type (infection *in.* or recovery *re.*), j_e is the affected individual, and t_j^I and t_j^R are the infection and recovery times for individual *j*, respectively.

Initialization: Each epidemic is assumed to be started by one (or potentially more) initially infected individual *j* at some initial time point t_{init}. The infection duration δt_j for this individual is drawn from a gamma distribution parameterised in terms of a mean and shape parameter:

$$\delta t_j \sim Gamma(w_j, k) \tag{A1}$$

(note, the dependency of w_j on θ is given through Eqs. (2) and (3)). This allows us to set $t_j^I = t_{init}$ and $t_j^R = t_{init} + \delta t_n$. Individual *j* is then placed onto a list $\mathcal{R}$, which represents all currently infected individuals. We set event index to *e=1*.

Step 1: Calculate the time to the next infection event. This is done by first evaluating the total transition rate that any individual becomes infected

$$\Lambda = \sum_s \lambda_s, \tag{A2}$$

where the sum *s* goes over all currently susceptible individuals and the force of infection λ_s (which gives the probability per unit time of *s* becoming infected) is given by Eq.(1). In accordance with a Poisson process, the time to the next infection event is generated by drawing a sample from the exponential distribution $\Lambda e^{-\Lambda\Delta t}$. In practice, this is achieved by selecting an inter-event time using

$$\Delta t = -\frac{\log(u)}{\Lambda}, \tag{A3}$$

where *u* is a (uniform) randomly generated number between *0* and *1*. The new event time is then defined by

$$t^{new} = t_{e-1} + \Delta t. \tag{A4}$$

Step 2: Choosing the event type. For the SIR model two possibilities exist:

a) If t^{new} is greater than the smallest recovery time of all the individuals in $\mathcal{R}$, which we label j_{min}, then we remove j_{min} from $\mathcal{R}$ and set

$$t_e = t_{j_{\min}}^R, \quad x_e = re., \quad j_e = j_{\min}. \tag{A5}$$

b) Otherwise, we set

$$t_e = t^{new}, \quad x_e = in., \tag{A6}$$

and select the actual individual that becomes infected with probability

$$\mathrm{Prob}(j_e = s) = \frac{\lambda_s}{\Lambda}. \tag{A7}$$

The infection duration δt_{j_e} for j_e is sampled using Eq.(A1), and the infection and recovery times are set to

$$\begin{aligned} t_{j_e}^I &= t_e, \\ t_{j_e}^R &= t_e + \delta t_{n_e}. \end{aligned} \tag{A8}$$

Individual j_e is then placed onto the list $\mathcal{R}$.

Step 3: Increment e and jump to step 1 if there are any remaining infected individuals.

End: Insert recovery times for any remaining individuals j in $\mathcal{R}$

$$t_e = t_j^R, \quad x_e = re., \quad j_e = j, \tag{A9}$$

incrementing e after each addition.

References

1. Gillespie DT. Exact Stochastic Simulation of Coupled Chemical-Reactions. J Phys Chem. 1977;81:2340-61.
