# Supplementary material for: Estimating individuals’ genetic and non-genetic effects underlying infectious disease transmission from temporal epidemic data
Source: PLoS Comput Biol. 2020 Dec 21;16(12):e1008447. doi: 10.1371/journal.pcbi.1008447 (PMC7785229; doi:10.1371/journal.pcbi.1008447)
Supplement: S7 Appendix — (PDF) [file pcbi.1008447.s007.pdf]

S7 Appendix: Infectivity SNP information coming from epidemic speeds

The keen observed may wonder how differences in epidemic speed can provide any information regarding the infectivity of individuals in contact groups. After all, the model contains a group effect term G_z in Eq.(1) which allows for group specific variations in transmission and surely this would absorb any differences. We now explain why this is not the case.

First it should be pointed out that the relative susceptibly of individuals is obviously a potential confounding factor. However, as mentioned in the main text, these can be independently calculated by looking at the *order* in which individuals become infected, so for the purposes of this discussion we will assume, without loss of generality, that all individuals have the same susceptibility.

Imagine a large number of contact groups that undergo epidemics, each with a different composition of SNP genotypes. Some of the epidemics are fast and some of them are slow, and some contain individuals with more *A* alleles and some with more *B* alleles. Suppose those groups with more *A* alleles tend to have faster epidemics. Do we conclude that the *A* allele confers greater infectivity or that this is just a random result caused by the group effect G_z? The answer is the former. In the Bayesian setting this can be explained because the parameter space is much larger in the former because the model requires just one model parameter a_f to be “tuned”, where as in the latter N_{group} group effects G_z must be tuned (hence whilst Bayesian analysis doesn’t preclude either option, there are many more posterior states in the former so they will tend to be sampled under MCMC).
