# Supplementary material for: Estimating individuals’ genetic and non-genetic effects underlying infectious disease transmission from temporal epidemic data
Source: PLoS Comput Biol. 2020 Dec 21;16(12):e1008447. doi: 10.1371/journal.pcbi.1008447 (PMC7785229; doi:10.1371/journal.pcbi.1008447)
Supplement: S8 Appendix — (PDF) [file pcbi.1008447.s008.pdf]

S8 Appendix: Parameter prediction accuracy under DS2

Fig S8 shows results when repeating the analysis is section 3.2 of the paper, but this time taking the scenario in which infection times are unknown (*i.e.* DS2). The corresponding regression analysis on these points is presented in Table S8. The average SDs in Table S8 are smaller than those in Table 1, reflecting the reduction in statistical power under this data scenario. A factor of two difference in SD corresponds to four times as many individuals required for equivalent accuracy.

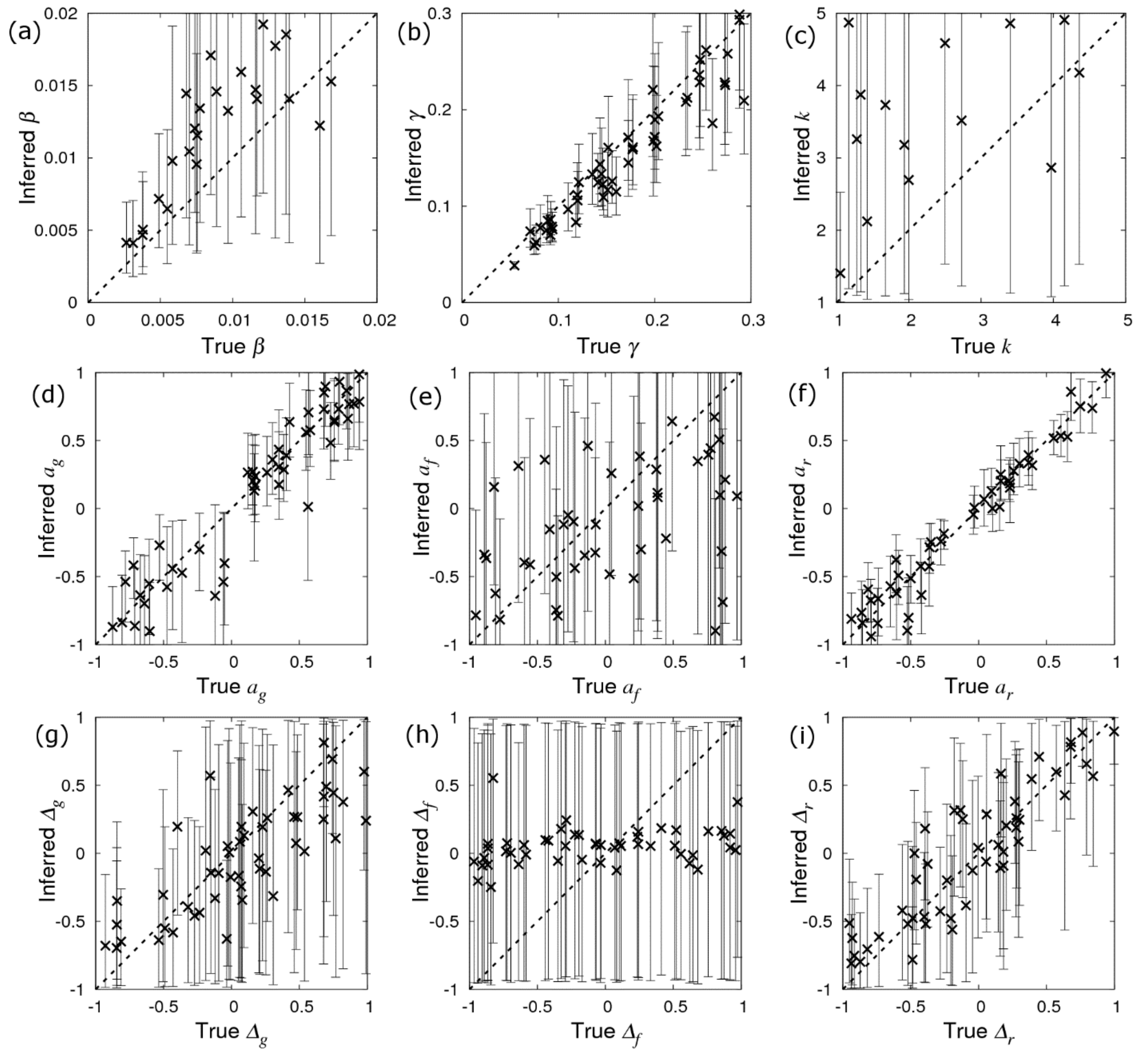

Fig S8. Prediction accuracy and bias. These plots summarise inferred posterior distributions for parameters compared to their true value. Simulated data was generated using the base parameter set in Eq.(10) except for a single parameter which was singled out in each of the sub-plots above*. Crosses correspond to the inferred posterior mean (with error bars indicating 95% credible intervals) of the selected parameter (whose true value is on the x-axis) when SIRE is applied to a single simulated data set consisting of recovery times (*i.e.* DS2) from N_{group}=20 contact groups each containing G_{size}=50 individuals. Prediction accuracies, and the intercept and slope of regression lines fitted to the data points are given in Table 1. (*Additionally for (g) a_g=0.4, (h) a_f=0.4 and (i) a_r=0.4, such that dominance has an effect).

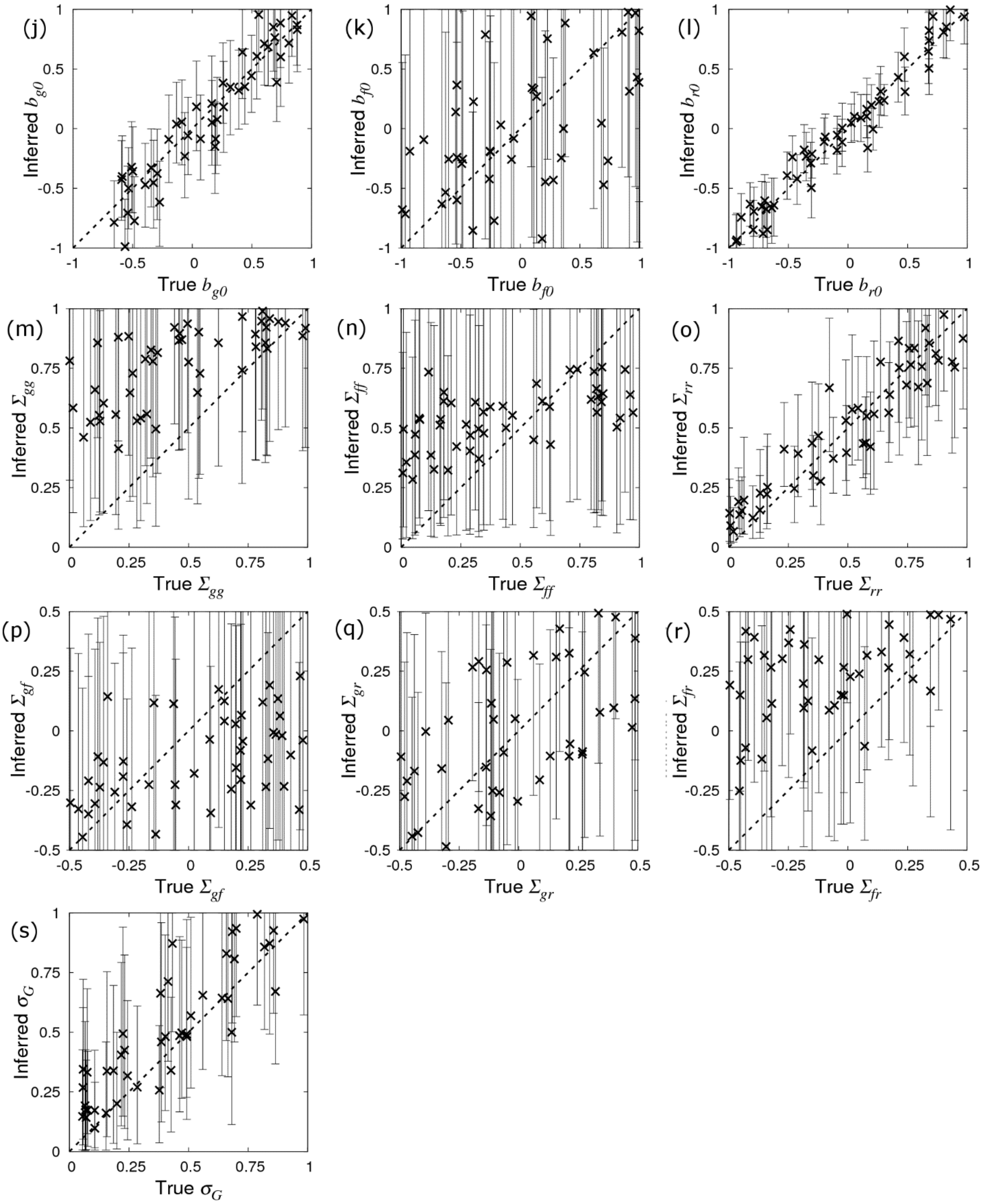

Fig S8 continued.

Parameter	Accuracy	*y*-intercept	Slope	Av. SD	Description
β	0.831	0.003	1.191	0.005	Average transmission rate
γ	0.956	-0.008	0.946	0.024	Average recovery rate
k	0.494	3.454	0.516	2.272	Recovery shape parameter
a_g	0.950	-0.004	0.995	0.173	SNP effect for susceptibility
a_f	0.355	-0.019	0.370	0.525	SNP effect for infectivity
a_r	0.979	-0.006	1.022	0.111	SNP effect for recoverability
Δ_g	0.791	-0.079	0.619	0.387	
Δ_f	0.260	0.056	0.054	0.544	Dominance factor (per trait)
Δ_r	0.891	-0.040	0.857	0.249	
b_{g0}	0.961	-0.033	1.083	0.188	
b_{f0}	0.411	-0.004	0.469	0.647	Fixed effect (per trait)
b_{r0}	0.978	0.011	0.980	0.111	
Σ_{gg}	0.723	0.581	0.416	0.236	
Σ_{ff}	0.503	-0.141	0.312	0.330	
Σ_{rr}	0.574	0.014	0.655	0.296	Residual covariance matrix
Σ_{gf}	0.588	0.440	0.233	0.266	
Σ_{gr}	0.537	0.292	0.382	0.248	
Σ_{fr}	0.941	0.101	0.817	0.113	
σ_G	0.906	0.131	0.936	0.182	SD of group effects

Table S8. Prediction accuracy and bias under DS2. Prediction accuracy is defined as the correlation between the inferred and true parameter values (a value of one implies perfect inference). The *y*-intercept and slope are taken from regression lines fitted through the data point (a *y*-intercept of zero and slope of one indicates no bias). Av. SD gives the average posterior standard deviation across all datasets.
