# Supplementary material for: Estimating individuals’ genetic and non-genetic effects underlying infectious disease transmission from temporal epidemic data
Source: PLoS Comput Biol. 2020 Dec 21;16(12):e1008447. doi: 10.1371/journal.pcbi.1008447 (PMC7785229; doi:10.1371/journal.pcbi.1008447)
Supplement: S9 Appendix — (PDF) [file pcbi.1008447.s009.pdf]

S9 Appendix: Computational speed estimate for SIRE

Fig S9 shows the CPU time SIRE takes to estimate the SNP effects as a function of the total number of individuals (this is based on a single 2GHz core). We find that the SNP effect associated with infectivity takes the longest to accurately estimate. The approximate linear scaling (represented by the solid black lines) means that SIRE is expected to take around one minute per 1000 individuals to generate 100 representative samples from the posterior under DS1 and around 10 minutes for DS2.

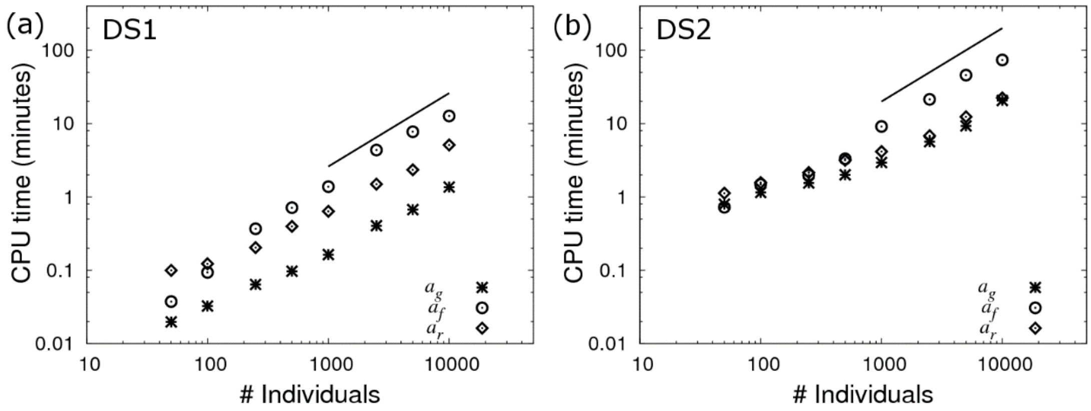

Fig S9. SIRE speed. The CPU time taken for SIRE to generate 100 independent posterior samples for the SNP effects as a function of the number of individuals (where the group size is taken to G_{size}=50), as estimated using the effective sample size [1]. Simulated data was generated using the base parameter set in Eq.(10) with (a) known infection and recovery times (DS1) and (b) known recovery times (DS2).
