# Supplementary material for: Estimating individuals’ genetic and non-genetic effects underlying infectious disease transmission from temporal epidemic data
Source: PLoS Comput Biol. 2020 Dec 21;16(12):e1008447. doi: 10.1371/journal.pcbi.1008447 (PMC7785229; doi:10.1371/journal.pcbi.1008447)
Supplement: S10 Appendix — (PDF) [file pcbi.1008447.s010.pdf]

## S10 Appendix: Polygenic contribution

In this appendix we test the assumption that ignoring polygenic contributions to host susceptibility, infectivity and recoverability does not affect SNP effect estimates for these traits, when individuals are randomly distributed across epidemic groups. This is achieved by adding an additive genetic contribution to the model when simulating data and then performing inference without such a term to see if it biases or alters the estimated SNP effects in any way.

For the simulations Eq.(3) in the paper becomes

$$\begin{aligned} \boldsymbol{g} &= \mathbf{X}\boldsymbol{b}_g + \boldsymbol{a}_g + \boldsymbol{\varepsilon}_g, \\ \boldsymbol{f} &= \mathbf{X}\boldsymbol{b}_f + \boldsymbol{a}_f + \boldsymbol{\varepsilon}_f, \\ \boldsymbol{r} &= \mathbf{X}\boldsymbol{b}_r + \boldsymbol{a}_r + \boldsymbol{\varepsilon}_r, \end{aligned} \tag{A1}$$

where $\boldsymbol{a}=(\boldsymbol{a}_g, \boldsymbol{a}_f, \boldsymbol{a}_r)$ accounts for the relationships in trait values between different individuals (these are multivariate-normally distributed with zero mean and covariance matrix[1] $\mathbf{A}\otimes\boldsymbol{\Omega}$, where **A** is the pedigree/genomic relationship matrix and $\boldsymbol{\Omega}$ is a 3×3 covariance matrix that characterises potential correlations between traits).

To take a simple case we imagine $N_{group}$=50 epidemic groups each containing $G_{size}$=20 individuals. To represent a relatively high degree of relatedness we randomly assign each individual to belong to one of 10 unrelated families with a full-sib structure (hence $A_{ij}$=0.5 if individuals $i$ and $j$ are in the same family, otherwise $A_{ij}$=0). The residual and additive genetic correlation matrices are taken to be

$$\Sigma = \sigma_e^2 \begin{pmatrix} 1 & 0 & 0 \\ 0 & 1 & 0 \\ 0 & 0 & 1 \end{pmatrix}, \qquad \Omega = \sigma_a^2 \begin{pmatrix} 1 & 0 & 0 \\ 0 & 1 & 0 \\ 0 & 0 & 1 \end{pmatrix}, \tag{A2}$$

where the total phenotypic variance $\sigma_P^2 = \sigma_e^2 + \sigma_a^2$ is fixed to one and the heritability $h^2 = \sigma_a^2/\sigma_P^2$ is varied.

Along with the choices in Eq.(A2), simulations were performed with a large SNP effect size on all three traits of $a_g$=$a_f$=$a_r$=0.5 and otherwise used the base set of parameters in Eq.(10). Inference was performed in just the same way as was used to derive Figs.(5-10) in the paper.

The results are shown in Fig S10. Here (a-c) gives the inferred values for the SNP effects (averaged over 50 simulated replicates). We see that they correspond well to the true value 0.5 (shown by the black line). As found previously, the variation about this true value (shown by the error bars) changes depending on the data scenario considered, with the best scenario DS1 (infection and recovery times exactly known) providing the least variation. Similarly, the precision of the SNP estimates in (d-f), as measured by the standard deviation in the posterior, is smallest for DS1 and goes up when either infection or recovery times are not known.

---

[1] The symbol "⊗" is a tensor product, *e.g.* the covariance between $a_{g,i}$ (the genetic contribution for susceptibility for individual $i$) and $a_{f,j}$ (the genetic contribution for infectivity for individual $j$) is given by $A_{ij}\Omega_{gf}$.

The important point to take from Fig S10 is that all the quantities show almost no variation with $h^2$. This means that even if there are substantial correlations in trait values between individuals, if those individuals are randomised across groups these correlations tend to average out so as to not bias the SNP effect estimates. Note, this additional uncertainty still acts to reduce precision, but in the same way as the residual environmental contribution allowing the two effects to be combined.

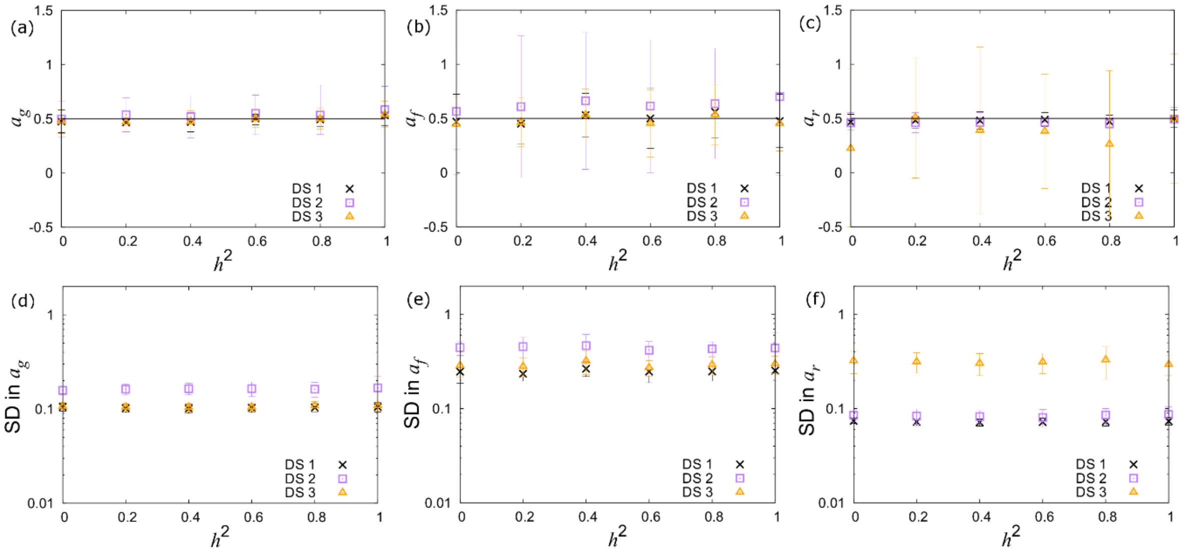


**Fig S10. SNP effect estimation and precision when traits truly heritable.** These plots shows how the inferred estimates for (a-c) SNP effects and (d-f) precisions vary as a function of the heritability $h^2$ when the inferred model ignores the underlying genetic correlations between individuals. Each symbol represents the average over 50 simulated data replicates with the error bar denoting 95% of the stochastic variation about this value. Different symbols represent different data scenarios: DS1) both the infection and recovery times for individuals are known, DS2) only recovery times are known, and DS3) only infection times are known.
