# Supplementary material for: Estimating individuals’ genetic and non-genetic effects underlying infectious disease transmission from temporal epidemic data
Source: PLoS Comput Biol. 2020 Dec 21;16(12):e1008447. doi: 10.1371/journal.pcbi.1008447 (PMC7785229; doi:10.1371/journal.pcbi.1008447)
Supplement: S11 Appendix — Information for users of the software tool. (PDF) [file pcbi.1008447.s011.pdf]

SIRE V1.0

# Susceptibility, Infectivity and Recoverability Estimation

Christopher M. Pooley[1,2*], Glenn Marion[2&], Stephen C. Bishop†, Richard I. Bailey[1]
and Andrea B. Doeschl-Wilson[1&]

[1] The Roslin Institute, The University of Edinburgh, Midlothian, EH25 9RG, UK.

[2] Biomathematics and Statistics Scotland, James Clerk Maxwell Building, The King's Buildings, Peter Guthrie Tait Road, Edinburgh, EH9 3FD, UK.

† Deceased

[&] These authors contributed equally to this work.

# Table of Contents


1 Introduction ........ 3

1.1 Downloading ........ 3

1.2 Getting started ........ 3

2 Inputs ........ 5

2.1 Description ........ 5

2.2 The data ........ 6

2.3 The model ........ 9

2.4 The priors ........ 12

3 Outputs ........ 13

3.1 Starting inference ........ 13

3.2 Trace plots ........ 14

3.3 Probability distributions ........ 16

3.4 Scatter plot ........ 16

3.5 Individual timelines ........ 17

3.6 Statistics ........ 17

3.7 Population plots ........ 19

3.8 Exporting ........ 19

3.9 Loading and saving ........ 20

4 Examples ........ 20

4.1 SIR model ........ 20

4.2 SI model ........ 21

5 Code ........ 21

6 License and warranty ........ 22

7 Citing SIRE ........ 22

8 Plans for SIRE v2.0 ........ 22

9 Acknowledgments ........ 22

References ........ 22

# 1 Introduction

Three key epidemiological host traits affect infectious disease spread: susceptibility (propensity to acquire infection), infectivity (propensity to transmit infection to others, once infected) and recoverability (propensity to recover quickly). SIRE is a desktop application for estimating factors affecting these three traits.

The term "contact group" refers to individuals sharing the same environment, such as a pasture, pen, cage, tank or pond. SIRE applies to individual-level disease data originating from one or more contact groups in which infectious disease is transmitted from infectious to susceptible individuals through effective contact (for simplicity it is assumed that groups are closed, *i.e.* no births, migrations, or disease transmission between them). Data can come from well controlled disease transmission experiments or from much less well controlled field data (which may be less complete, but readily available in greater quantity).

SIRE takes as input any combination of information about infection times, recovery times, disease status measurements, disease diagnostic test results, genotypes at a particular SNP[1] under investigation (if available), other fixed effects, details of which individuals belong to which contact groups and any prior specifications. The output from SIRE consists of posterior trace plots for model parameters, distributions, visualisation of infection and recovery times, dynamic population estimates and summary statistics (means and 95% credible intervals) as well as MCMC diagnostic statistics.

A detailed description of the epidemiological and statistical model underlying SIRE along with the Bayesian inference methodology is given in an accompanying paper [1]. The focus of this manual is on the practicalities of analysing real world data and interpreting the results.

## 1.1 Downloading

SIRE is freely available under the GNU General Public License, and can be downloaded from www.mkodb.roslin.ed.ac.uk/EAT/SIRE.html.

Depending on your platform, the following instructions explain how SIRE can be run:

- **Windows** – Download the file "SIRE_v1.0_windows.zip" and unzip. SIRE is run by clicking on the "SIRE.exe" icon.
- **Linux** – Download the file "SIRE_v1.0_linux.tar.gz". This can then be extracted by using the terminal command "tar -zxvf SIRE_v1.0_linux.tar.gz". The code is executed using "./SIRE".
- **Macintosh** – Download the file "SIRE_v1.0_Mac.zip". SIRE is run by clicking on the "SIRE.app" icon[2].

## 1.2 Getting started

Figure 1 shows the screen displayed when SIRE is first loaded. The main menu on the left (Fig. 1A) is used to navigate arbitrarily from page to page. To begin three options are available: a previous

---

[1] SNP stands for "single nucleotide polymorphism" and refers to particular locations along the genome that exhibit a large degree of variability across the population.

[2] If the error message "SIRE can't be opened because it is from an unidentified developer…" appears, right clicking on "SIRE.app" and selecting "Open" will allow the option to run.

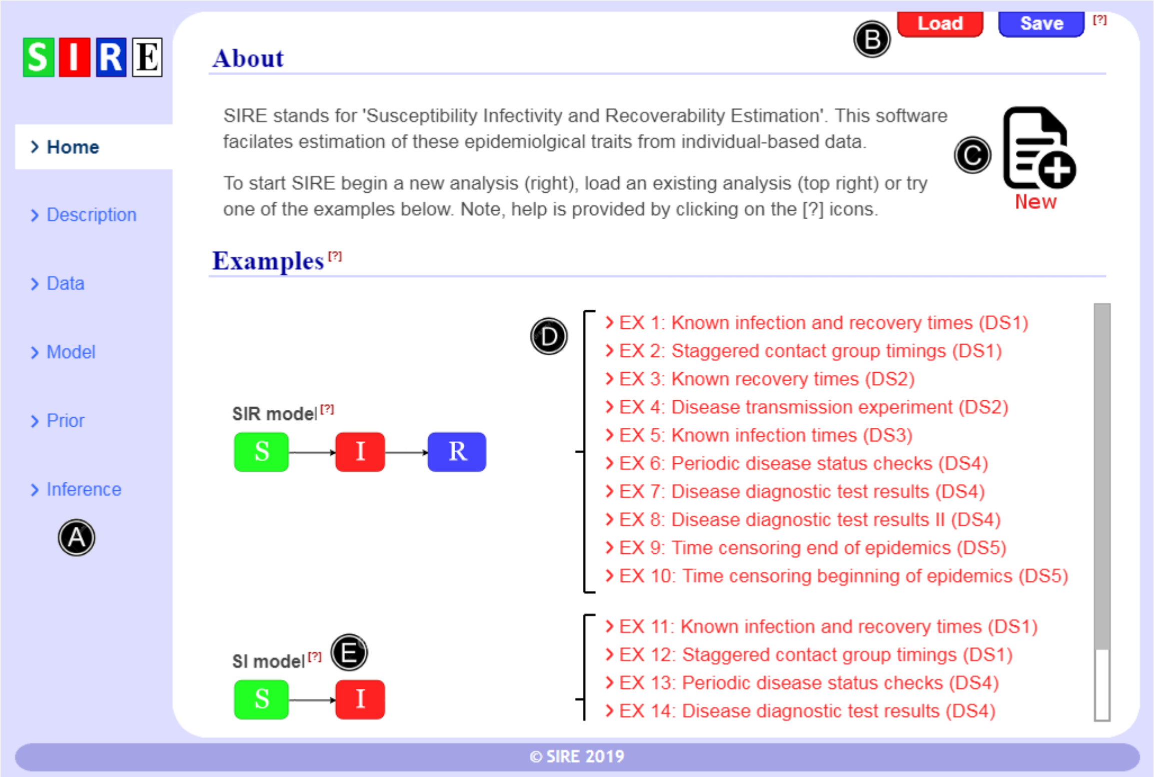


**Figure 1** – *The home screen*. A: Main menu, B: load previous analysis, C: start a new analysis, D: examples looking at various data scenarios (DS), E: Click on [?] for more information.

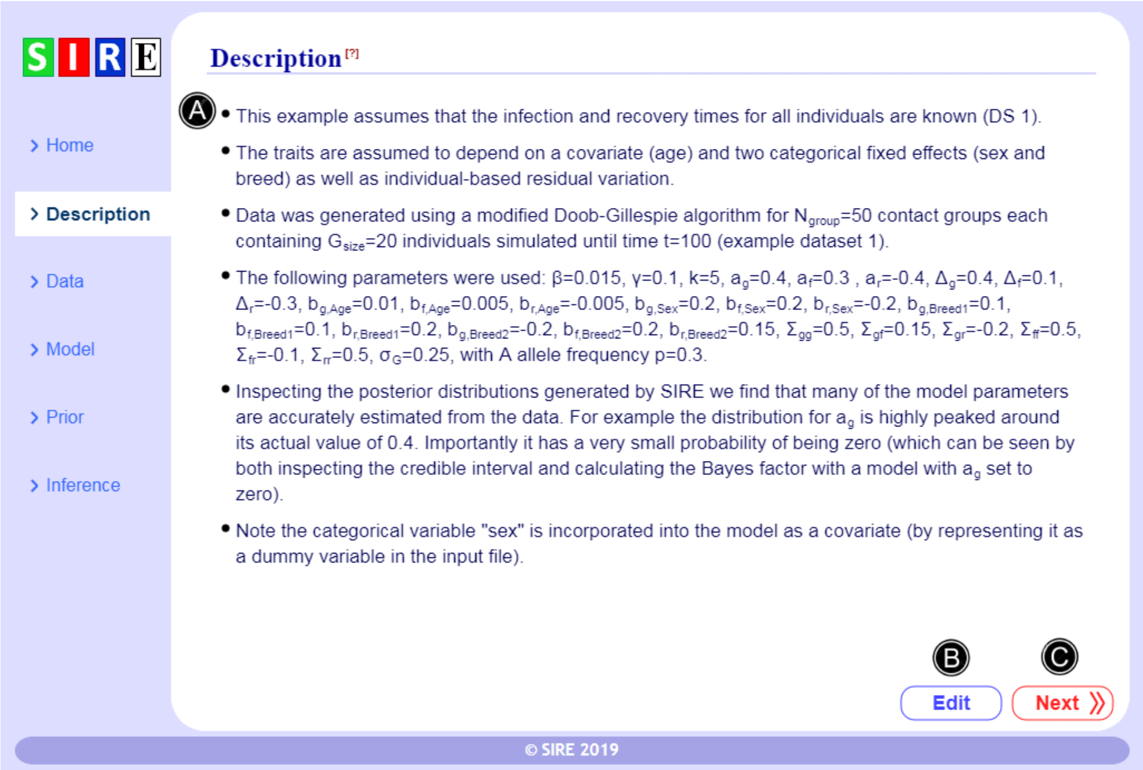


**Figure 2** – *Description*. A: Text panel showing a description of the data and model assumptions (here for EX.1); B: edit this description, C: go to the next step. Note, definitions for the parameters mentioned above are given in Table 1, and further details of the model are provided in section 2.3.

analysis can be loaded (Fig. 1B, note SIRE uses a special ".sire" file format for loading and saving analyses, as described in section 3.9), a new analysis can be started (Fig. 1C), or one of the illustrative examples can be investigated (Fig. 1D). The examples refer to different data scenarios (DS) that SIRE can handle (see section 4). New users are encouraged to try these first and spend some minutes exploring the software to get a feeling for how it works. Successively clicking "Next" goes through the various data and model options (discussed in detail below), and starting inference leads to several visualisations for the posterior. These examples can be modified (*e.g.* by making changes to the model/data), but the default settings are restored when reloaded from the home screen in Fig. 1.

Additional information on many of the screens can be gained by clicking on the [?] buttons (*e.g.* Fig. 1E). This manual follows the order of the items on the main menu (Fig. 1A).

# 2 Inputs

This section describes how data is incorporated into SIRE, along with specification of the model and prior[3].

## 2.1 Description

As shown in Fig. 2A, SIRE allows users to provide a brief description of the data and assumptions used for analysis. This is not only useful to keep track for personal use, but also makes it easier and more transparent for others to understand what has been done. The description can be edited by clicking on Fig. 2B (note, bullet points are automatically generated for each carriage return in the editable text box). Complete these details and click "Next" at the bottom right of the screen (Fig. 2C). Note, the next button on each of the pages is just for convenience. In fact the menu on the left hand side of the screen can be used to arbitrarily navigate to any page without loss of information.

| | A | B | C | D | E | F | G | H | I |
|---|---|---|---|---|---|---|---|---|---|
| 1 | ID | Group | SNP | It | Rt | Age | Sex | Breed | Type |
| 2 | ind0 | Gr 1 | AB | 0 | 8.52943 | 39 | 1 | Breed 1 | Seeder |
| 3 | ind1 | Gr 1 | AB | 7.46777 | 17.5333 | 36 | 0 | Breed 2 | Contact |
| 4 | ind2 | Gr 1 | AA | 3.26406 | 44.8453 | 39 | 0 | Breed 3 | Contact |
| 5 | ind3 | Gr 1 | AA | 3.02582 | 12.8424 | 49 | 0 | Breed 2 | Contact |
| 6 | ind4 | Gr 1 | AA | 3.7433 | 18.6858 | 37 | 0 | Breed 3 | Contact |
| 7 | ind5 | Gr 1 | AB | 3.84091 | 12.0541 | 32 | 0 | Breed 1 | Contact |
| 8 | ind6 | Gr 1 | AA | 4.53991 | 21.1796 | 40 | 1 | Breed 3 | Contact |
| 9 | ind7 | Gr 1 | AB | 3.93424 | 9.49594 | 33 | 0 | Breed 3 | Contact |
| 10 | ind8 | Gr 1 | AB | 2.73098 | 17.4823 | 20 | 0 | Breed 1 | Contact |
| 11 | ind9 | Gr 1 | AA | 12.3191 | 23.9068 | 33 | 1 | Breed 2 | Contact |
| 12 | ind10 | Gr 1 | AB | 3.55243 | 11.9158 | 22 | 1 | Breed 2 | Contact |
| 13 | ind11 | Gr 1 | AB | 3.2593 | 12.9687 | 44 | 0 | Breed 2 | Contact |
| 14 | ind12 | Gr 1 | AA | 3.75865 | 20.2027 | 44 | 0 | Breed 1 | Contact |
| 15 | ind13 | Gr 1 | AB | 3.24161 | 6.01077 | 32 | 0 | Breed 3 | Contact |
| 16 | ind14 | Gr 1 | AB | 2.00271 | 50.6246 | 47 | 1 | Breed 3 | Contact |
| 17 | ind15 | Gr 1 | AA | 2.07932 | 19.6202 | 21 | 1 | Breed 1 | Contact |
| 18 | ind16 | Gr 1 | AB | 9.02055 | 32.4941 | 28 | 1 | Breed 3 | Contact |
| 19 | ind17 | Gr 1 | AA | no | no | 40 | 1 | Breed 3 | Contact |
| 20 | ind18 | Gr 1 | AA | 3.386 | 8.68745 | 43 | 1 | Breed 2 | Contact |
| 21 | ind19 | Gr 1 | AA | 32.4559 | 48.4037 | 27 | 0 | Breed 1 | Contact |
| 22 | ind20 | Gr 2 | AB | 0 | 4.06551 | 29 | 0 | Breed 1 | Seeder |
| 23 | ind21 | Gr 2 | AA | 15.5408 | 46.6454 | 41 | 1 | Breed 2 | Contact |
| | | ⋮ | | ⋮ | | | ⋮ | | |

**Figure 3**– *Data formatting*. This shows an example data table from a simulated disease transmission experiment. The columns are defined as ID: a unique identifier for individuals, Group: the name of the contact group that individual belongs to, SNP: the genotype at a particular SNP, It: the observed infection time, Rt: the observed recovery time, Age: the age, Sex: where 1 implies male, Breed: breed of the individuals, and Type: set as "Seeder" if an individual is infected at the beginning of the transmission trial else "Contact".

[3] Priors encapsulate previous knowledge regarding model parameters and ensure they are confined to physically realistic values during Bayesian inference.

## 2.2 The data

First we describe how data needs to be formatted to be imported into SIRE. Figure 3 shows an example dataset (in fact the one used for EX.1), as displayed on a spreadsheet. SIRE is flexible in terms of reading in different types of data, and so the user's data doesn't necessarily need to look like this. Importantly, however, SIRE does rely on one column giving individual IDs. Other columns can relate to whatever data happens to be available (with possibly extraneous columns, such as "Type" in this particular example, which are ignored), details of which are discussed later. The spreadsheet needs to be saved in .csv or tab-delimited .txt format so that it can be read by SIRE (an example of this is the "Dataset 1.txt" file in the Datasets directory of SIRE).

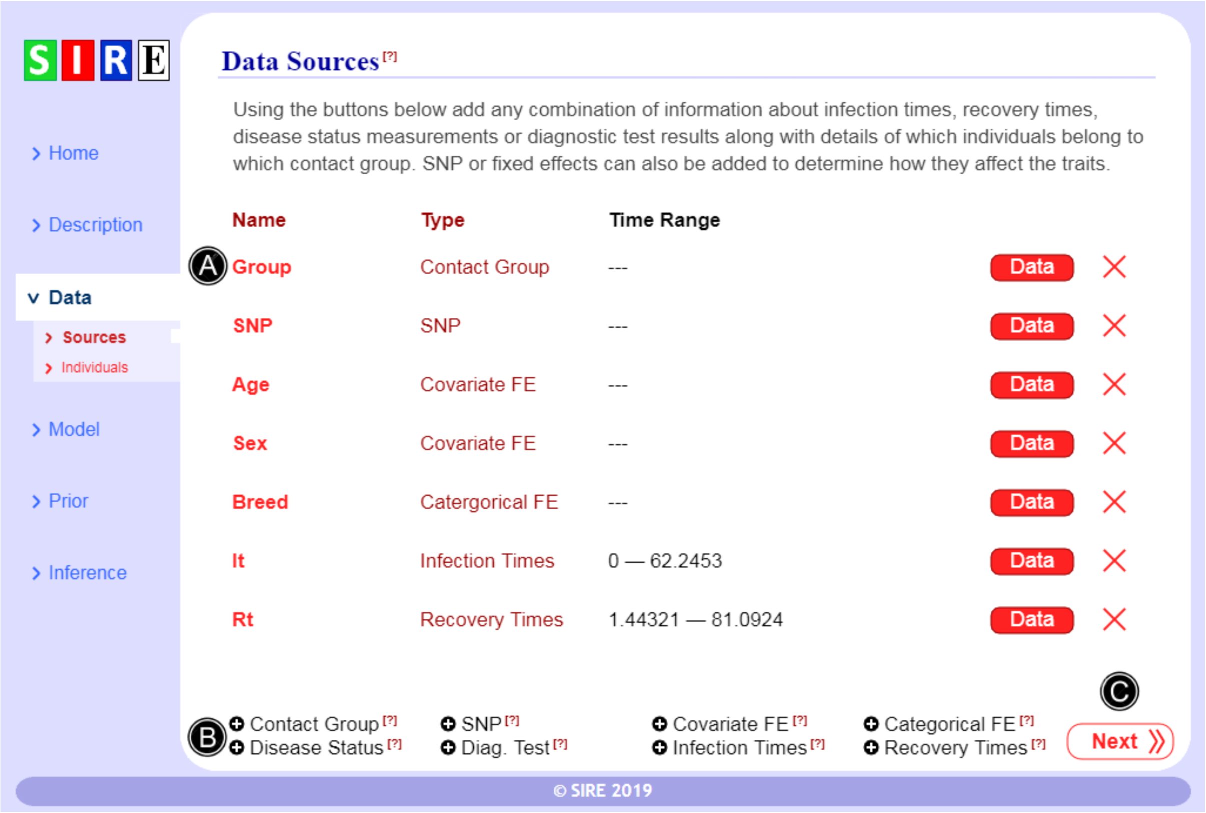


**Figure 4**– *Inputting data*. A: List of data sources, B: add new types of data, C: go to next page.

Next we discuss how information from this file is incorporated into SIRE. Rather than loading all the data at once, the user loads different sources of data, one at a time (in any order). A summary of the data sources loaded for EX.1 is shown in Fig. 4A. This shows information is available about which individuals are in which contact groups, various fixed effects and the infection and recovery times for all individuals over a defined observation period.

Clicking on the buttons in Fig. 4B allows for various types of data to be added (whichever appropriate):

- **Contact group** – Determine which individuals belong to which contact group. If this data is absent, it is assumed that all individuals share the same contact group.
- **SNP** – Provide the genotypes of individuals at a particular SNP under investigation (these must take one of the following possibilities: "AA", "AB", "BA" or "BB").

- **Covariate fixed effect (FE)** – Provide numerical covariate data (*e.g.* age) which potentially modifies the three epidemiological trait values. Here the outputted fixed effects represent regression slopes relating the traits to the data. Note, this approach can also be used to represent binary traits, *e.g.* 1 or 0 elements representing male or female (in which case the fixed effects represent sex-based differences in the traits).
- **Categorical fixed effect (FE)** – Data consisting of the category to which an individual belongs (*e.g.* breed). A reference category is selected (later) and the fixed effects represent the fractional change in the three epidemiological traits compared to this reference.
- **Disease status** – Data giving the infection status of individuals at particular points in time (these must take one of the following possibilities: “S”, “I”, “R” or “.” if unknown).
- **Diagnostic test results** – Diagnostic test results at particular points in time (these must take the values “1” or “0” corresponding to positive or negative test results or “.” if unknown). Additionally, the test has an associated sensitivity *Se* and specificity *Sp* which must be set. Note, tests can be selected to be sensitive to both the *I* and *R* states (*e.g.* appropriate for a serological test), or just the *I* state (*e.g.* appropriate for a culture test). Multiple sets of results from different diagnostic tests (*e.g.* ELISA/ γ-interferon / culture) can be incorporated into a single analysis.
- **Infection times** – Provide the times at which individuals become infected. If no infection is observed then the entry “no” is used and if not known then “.” is used. The range in time over which observations are actually made (allowing for censoring) is given in section 3.1.
- **Recovery times** – Provide the times at which individuals recover (or die in the case of disease induced mortality). If no recovery is observed then the entry “no” is used and if not known then “.” is used. Again, the observation time range is given in section 3.1.

Note, not all of these data types are needed. For example in some cases only the death times of individuals are known (which are inputted as 'Recovery Times'), or in other scenarios only diagnostic test results are available. These various options are explored in the examples. Data can be viewed or edited by clicking on the red buttons in Fig. 4A, or deleted by clicking on the corresponding red crosses.

As noted above, many sources of data include a missing data option (represented by “.”). However for the current version of SIRE, specified contact group, SNP and FE information is assumed to be precisely known.

After one of the possibilities in Fig. 4B is clicked, the user will be prompted to load the data file containing the required information, which will be the .txt or .csv file saved from the users spreadsheet. Once loaded the table is displayed on the screen, an example of which is shown in Fig. 5A. The user is prompted to select the key columns (in this example contact group information is being added) and click “Done” when complete. SIRE also provides some basic data editing capabilities (Fig. 5B). These allow searching and replacing (useful, for example, when converting “+” and “-“ to “1” and “0” for diagnostic test results), sorting and deleting (*e.g.* for removing missing data).

Once all the data sources have been added “Next” (Fig.4C) is clicked, which allows the user to view the data using the “Individuals” tab (Fig. 6). This shows timelines summarising temporal data for each of the individuals as well as contact group, SNP and fixed effect information.

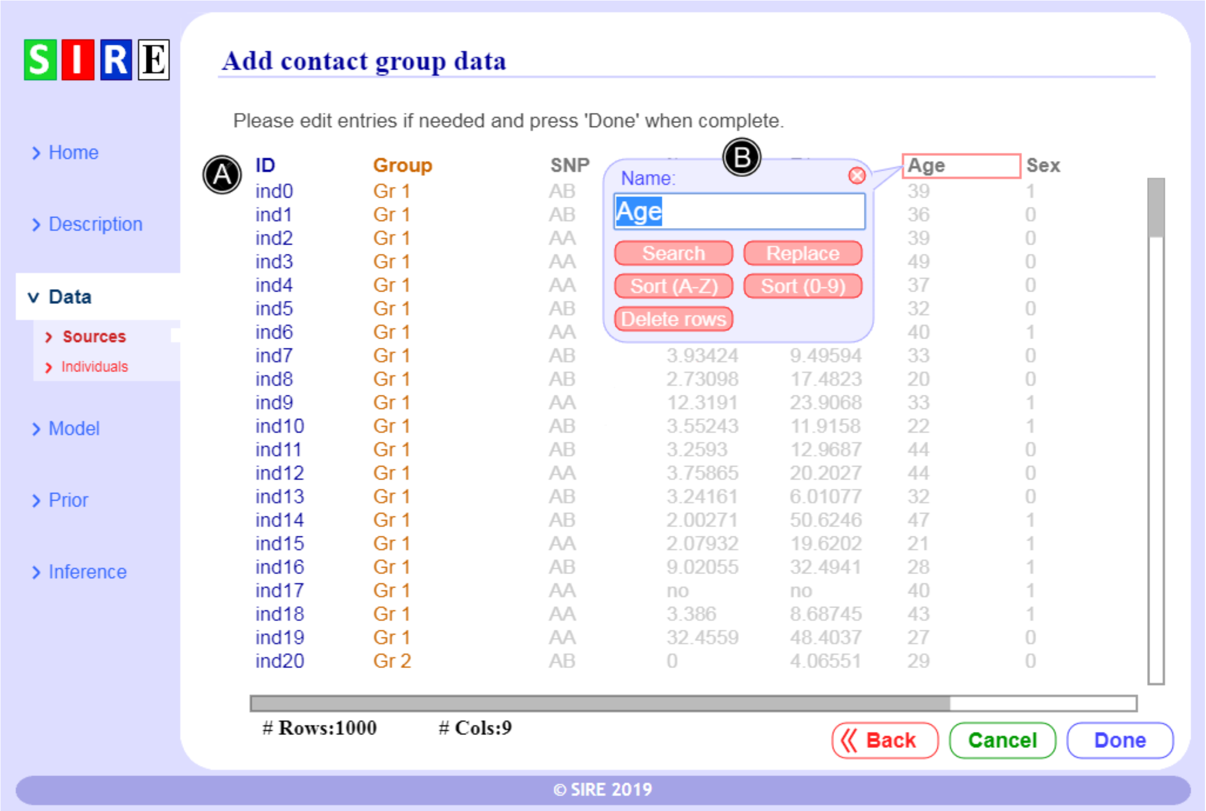


**Figure 5** – Inputting *data tables*. A: Tables are loaded in .txt or csv format and the relevant data is then extracted, B: basic editing/manipulation of data can be performed.

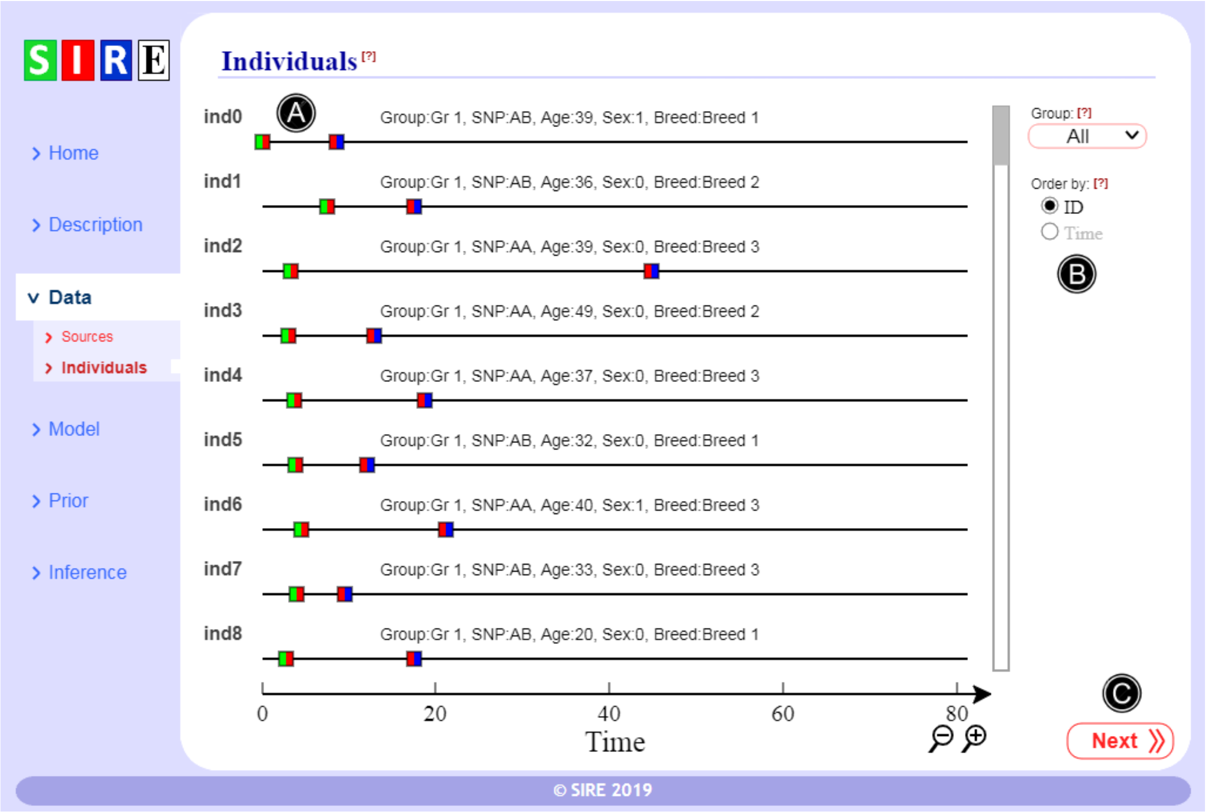


**Figure 6** – *Viewing data*. A: Individual-based data (green/red or red/blue squares give infection and recovery times), B: filter by group and ordered by ID or time of first observation, C: go to next page.

## 2.3 The model

| Type | Parameter | Description |
|---|---|---|
| *Epidemiological* | $\beta$ | Population average contact rate. |
| | $\gamma$ | Population average recovery rate. |
| | $k$ | Shape parameter that characterises the gamma distributed infection duration. |
| *Genetic* | $a_g$,$a_f$, $a_r$ | SNP effects, *i.e.* fractional change in susceptibility, infectivity or recoverability coming from an *A* allele compared to a *B* allele. |
| | $\Delta_g$,$\Delta_f$,$\Delta_r$ | Corresponding scaled dominance factors (1 when *A* is completely dominant over *B*). |
| *Fixed effects* | $\mathbf{b}_g$,$\mathbf{b}_f$,$\mathbf{b}_r$ | Vectors of fixed effects for the three traits. |
| *Residuals* | $\boldsymbol{\varepsilon}_g$, $\boldsymbol{\varepsilon}_f$, $\boldsymbol{\varepsilon}_r$ | Residual contributions to the traits (that is individual-based variation over and above that coming from the SNP, fixed effects or group effects). |
| | $\boldsymbol{\Sigma}$ | 3x3 covariance matrix for residual contributions. This accounts for potential correlations between the residuals for each traits (*e.g.* more susceptible individuals may also be more infectious). |
| *Group effects* | $G_z$ | Group effects (accounts for fractional differences in transmission rates in different groups). |
| | $\sigma_G$ | Standard deviation in group effects. |

**Table 1.** This gives a brief description of all the (potential) parameters in the model.

Here we describe how there model parameters are incorporated into the underlying epidemiological and statistical model used to analyse the data. SIRE generally assumes that the process of disease spread within a contact group follows an epidemiological SIR or SI model, where individuals' transition rates may be affected by various systematic (*e.g.* genotypic) and random effects. A comprehensive description is given in [1] which we briefly reiterate here for convenience. For the SIR model individuals are classified as being either susceptible to infection (*S*), infected and infectious (*I*), or recovered/removed/dead (*R*). The time-dependent force of infection for a susceptible individual *j* (*i.e.* the probability per unit time of becoming infected) is given by $\lambda_j$(t). For those individuals that do become infected, the duration over which they are infectious is assumed to be gamma distributed with mean $w_j$ and shape parameter *k*. These quantities can be expressed in the following way:

$$\lambda_j(t) = \beta e^{G_z} e^{g_j} \sum_i e^{f_i}, \qquad w_j = (\gamma e^{r_j})^{-1}, \tag{1}$$

where β and γ are population average transmission and recovery rates and $G_z$ is a so-called "group effect" (*z* indexes contact group) that accounts for group-specific factors that influence the overall speed of an epidemic in one contact group relative to another (*e.g.* animals kept in different management conditions, environmental differences, or variation in pathogen strains with different virulence). $G_z$ is assumed to be a random effect with standard deviation $\sigma_G$.

In Eq.(1), $g_j$ characterises the fractional deviation[4] in individual *j's* susceptibility as compared to that of the population as a whole, $f_i$ characterises the corresponding quantity for individual *i's* infectivity, and $r_j$ relates to recoverability. These, themselves, can be decomposed into various factors

$$\begin{aligned} \boldsymbol{g} &= \boldsymbol{g}^{\mathrm{SNP}} + \boldsymbol{X}\boldsymbol{b}_g + \boldsymbol{\varepsilon}_g, \\ \boldsymbol{f} &= \boldsymbol{f}^{\mathrm{SNP}} + \boldsymbol{X}\boldsymbol{b}_f + \boldsymbol{\varepsilon}_f, \\ \boldsymbol{r} &= \boldsymbol{r}^{\mathrm{SNP}} + \boldsymbol{X}\boldsymbol{b}_r + \boldsymbol{\varepsilon}_r, \end{aligned} \tag{2}$$

which are, respectively, a SNP effect (which may or may not be in the model depending on whether SNP data is available), fixed effects (such as age, sex or vaccination status) and residual variation. The SNP effect itself is dependent on the genotype of individuals (taken to be *AA*, *AB* or *BB* for diploid organisms, where *A* and *B* are two potential alleles at the genetic locus under investigation) in the following way

$$\left.\begin{matrix} & a_g & & a_f & & a_r \\ {}_j^{SNP} = & a_g\Delta_g, & f_j^{SNP} = & a_f\Delta_f, & r_j^{SNP} = & a_r\Delta_r \\ & -a_g & & -a_f & & -a_r \end{matrix}\right\} \begin{matrix} \text{if } j \text{ is } AA \\ \text{if } j \text{ is } AB \\ \text{if } j \text{ is } BB \end{matrix} \tag{3}$$

The parameters $a_g$, $a_f$ and $a_r$ capture the relative differences in trait values between *AA* and *BB* individuals and the scaled dominance factors $\Delta_g$, $\Delta_f$ and $\Delta_r$ characterise the trait deviations between the heterozygote *AB* individuals and the homozygote mean (a value of 1 corresponds to complete dominance of the *A* allele over the *B* allele and -1 when the reverse is true).

[4] *E.g.* $g_j$=0.1 corresponds to individual *j* being ≃10% more susceptible than the population average.

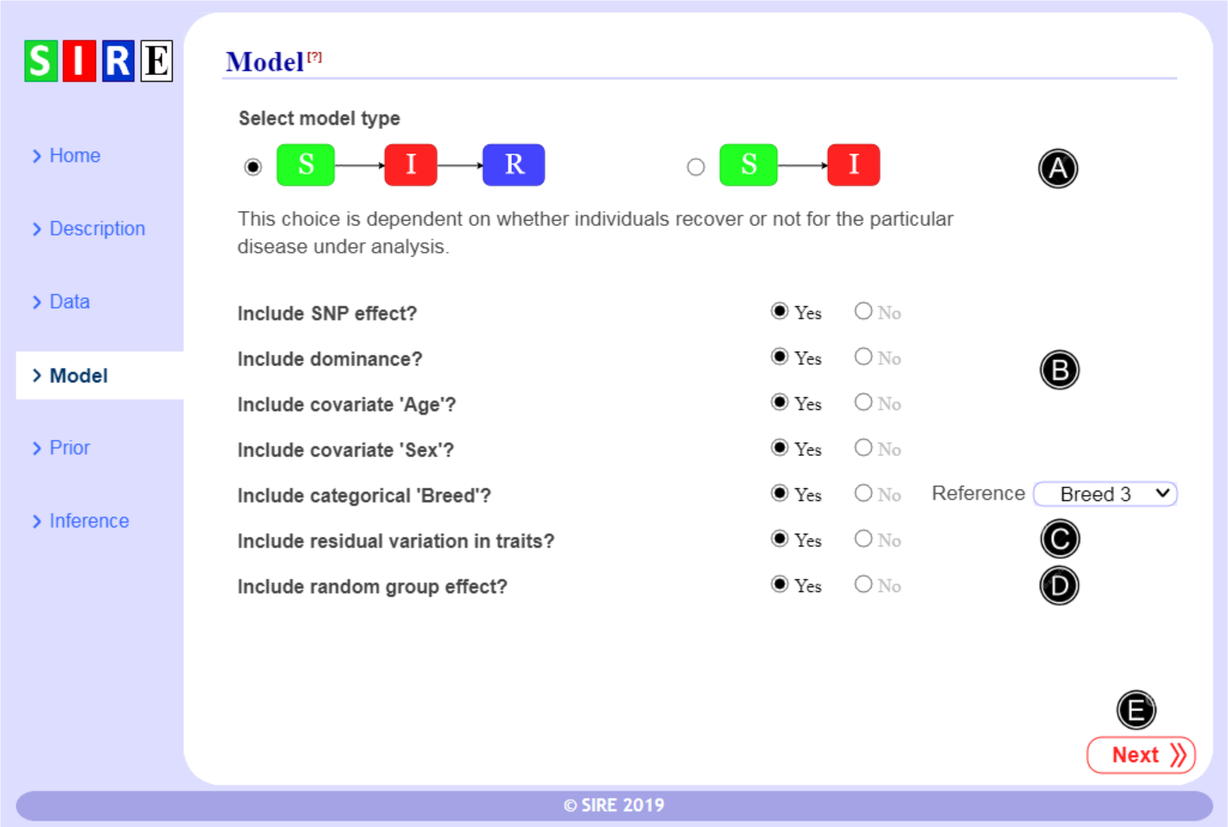

**Figure 7** – *Selecting the model.* A: Choose depending on whether recoveries or mortalities occur or not, B: choose which SNP or fixed effects to include (note for categorical fixed effects the reference needs to be specified), C: include residual variation in traits above and beyond contributions coming from SNP/fixed/group effects, D: include random group effect or not, E: go to next page.

Various features of the model outlined above can be altered:

- Figure 7A determines the compartment model type. For some diseases individuals do not recover (*e.g.* bovine tuberculosis), so a simper SI model is more appropriate than the full SIR.
- Figure 7B allows for SNP (with or without dominance) or fixed effects to easily be turned on or off in the model.
- Figure 7C determines the inclusion or absence of the **ε** terms in Eq.(2). In most standard analyses this variation (*i.e.* in addition to individual-based variation coming from the SNP/fixed effects) is ignored. In reality, however, these contributions may play an important role is determining disease dynamic behaviour, and so it is recommended to keep this within the model (although in cases in which MCMC convergence is a problem switching these off can be beneficial).
- Figure 7D determines the inclusion or absence of the $G_z$ group effect term in Eq.(1). In a well-controlled disease challenge experiment, where extraneous factors are largely controlled, it may be appropriate to neglect group effects because their inclusion in the model leads to an unnecessary reduction in parameter precision. On the other hand for most real-world field data, environmental variation across different locations would doubtless lead to substantial variation in transmission rate, and so inclusion of $G_z$ becomes a necessity.

Click "Next" (Fig.7E) after completing model specification.

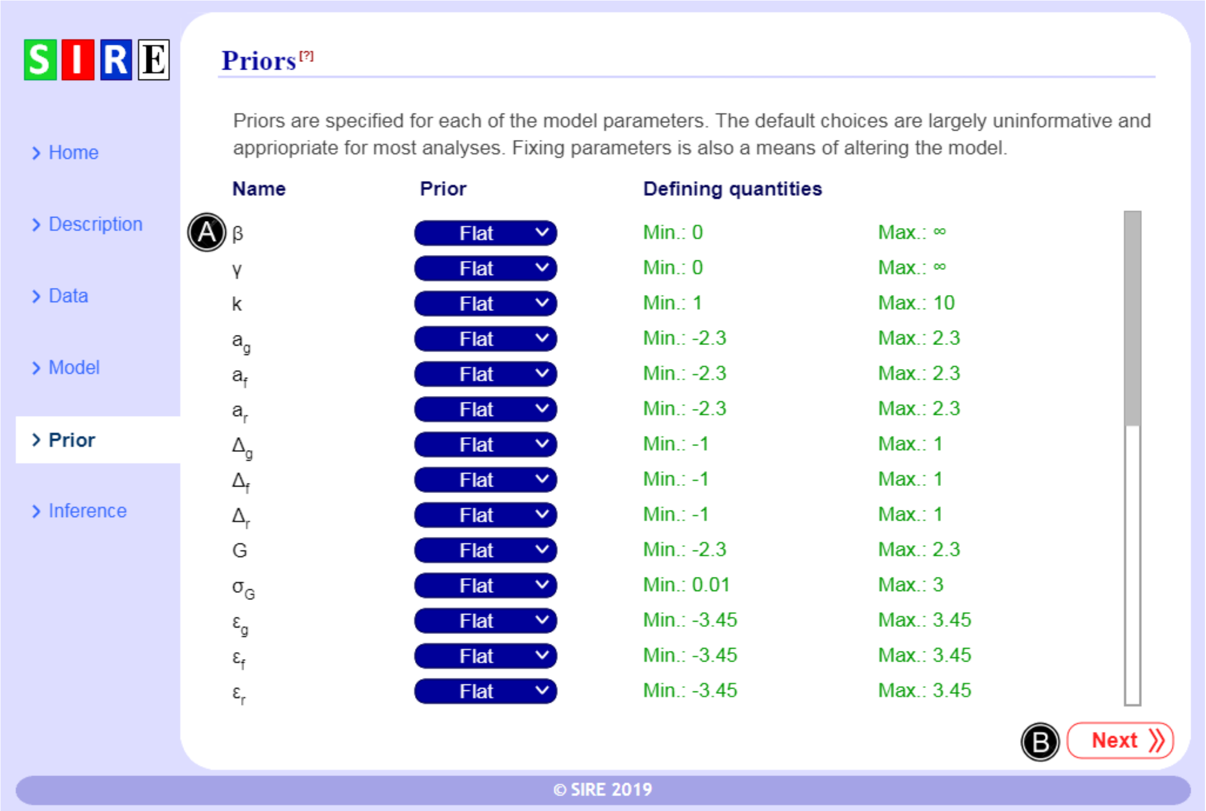


**Figure 8** – *Specification of the prior.* A: A list of all model parameters along with prior specifications (note parameters can also be fixed by selecting "Fix" from the drop-down menu). This example shows default values used in SIRE, B: go to the next page.

## 2.4 The priors

Priors are specified for each of the model parameters (see Table 1 for reference). The default choices are largely uninformative but do place upper and lower bounds on many of the key parameters (this stops them straying into biologically unrealistic regimes during inference, see appendix C in [1] for further details). Bounding parameters in this way is especially important when considering relatively uninformative data scenarios, when unbounded flat priors can lead to improper posterior probability distributions.

SIRE supports the following prior specifications: flat, which relates to a uniform probability distribution across a range, and the gamma, normal, log-normal and beta distributions, as well as the possibility to fix parameters to specific known values. Additionally, the prior can be chosen to return to its default setting.

It should be noted that the default settings in SIRE are generally suitable for most scenarios and changing them is usually only appropriate if specific knowledge on parameter values actually exists. However, fixing parameters is also a means of altering the model. For example setting shape parameter $k$=1 results in the model assuming exponentially distributed infection duration (*i.e.* it becomes Markovian).

Click "Next" (Fig. 8B) to complete the data and model section and move onto inference.

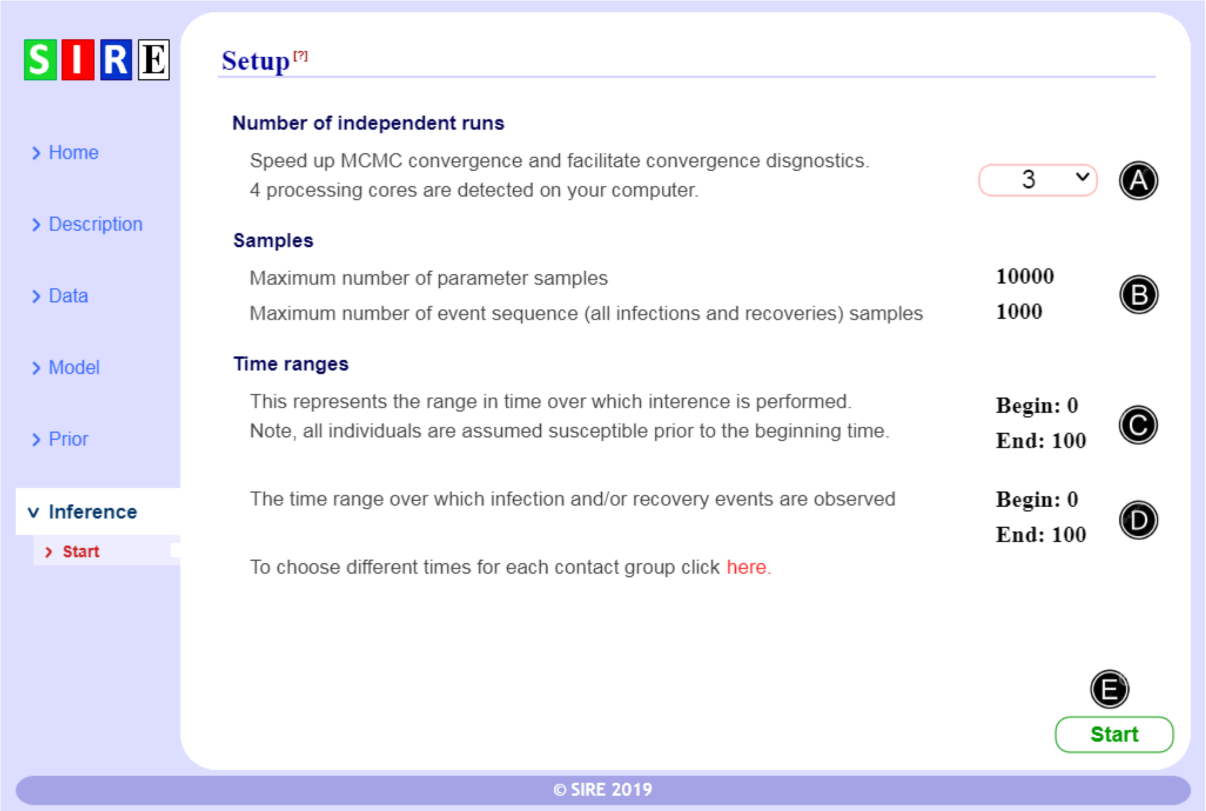


**Figure 9** – *Initialising inference.* A: Number of MCMC runs, B: limits the number of parameter and event sequence samples, C: inference time range, D: observation time range, E: start inference.

# 3 Outputs

Based on the data entered in section 2 it is generally not possible to identify model parameters with perfect precision (or infection and recovery times for that matter, unless they are specifically specified in the data). Rather there exists a distribution in these quantities known as the "posterior", which expresses both a best guess for parameters (*i.e.* posterior means) along with ranges in parameter values consistent with the data (*i.e.* credible intervals). SIRE achieves Bayesian inference by means of drawing samples from this posterior distribution using a widely used technique known as Markov chain Monte Carlo [2]. Unlike other statistical techniques (such as maximum likelihood) MCMC does not simply output a final answer. Rather it successively generates samples which progressively improve the accuracy of the posterior estimate until sufficient accuracy is achieved (how long this process takes is discussed in section 3.6).

## 3.1 Starting inference

Several options must be selected before inference can begin:

- The number of MCMC runs to be executed is selected using the drop-down menu at Fig. 9A. Each run exists on the computer as a separate process, and since most modern computers contain multiple CPU cores, computationally efficiency can be substantially improved by selecting more than one run[5]. Additionally, executing multiple runs allows for MCMC

[5] Executing more runs than the number of CPU cores can lead to a substantial slowing down of the SIRE interface, which is not recommended.

diagnostics to verify convergence (see section 3.6). A suitable choice is 3, which is used as the default value.

- As SIRE runs it collects parameters samples for both the model parameters and infection/recovery events for all the individuals in the population. Setting the values in Fig. 9B high allows for smoother output plots to be made, but can potential lead to insufficient computational memory. The default values represent a sensible trade-off between the two.
- Figure 9C shows the time range over which inference is performed. This must include the times at which data is actually collected, but may also be extended forward in time to allow for future model prediction. It is important to note here that at the “Begin” time SIRE assumes that all individuals are susceptible. Consequently in scenarios in which the initiation time of epidemics is unknown, this “Begin” time must be set significantly prior to the time at which data is collected.
- In cases in which infection and/or recovery data is available, it is necessary to identify the time range over which these observation are made (Fig. 9D). This allows for the possibility of time censoring whereby only the beginning or end of the epidemics are actually observed.
- Finally, if the time ranges for each epidemic group are different, this can be selected.

Bayesian inference can now begin by clicking on the “Start” button (Fig. 9E).

## 3.2 Trace plots

As seen in Fig. 10A, the first screen after inference is started shows trace plots for model parameters. As mentioned previously, MCMC works by successively drawing parameter samples (represented by the $x$-axis) from the posterior. Ideally these samples should be randomly distributed, but in reality they are correlated (which manifests itself by structure within these plots). The example in Fig. 10A is one in which mixing is good, because the curves exhibit substantial variation up and down about the posterior mean. Under different circumstances, however, MCMC runs can exhibit poor mixing, resulting in SIRE taking much longer to provide results adequately representative of the posterior. The examples in section 4 (which all consist of analysing 1000 individuals under different data scenarios) take from a few seconds to a few minutes to adequately mix. Measures for assessing how long inference should be performed are discussed in section 3.6.

Different parameters are selected in the following way: The drop-down menu (Fig. 10B) classifies different types of variable (“SNP” gives parameters related to SNPs, “Covar.” gives the covariance matrix, “Epi.” gives epidemiological parameters, “Gr. Eff.” gives the group effects, “Fix. Eff.” gives any fixed effects, and “Misc.” gives any other quantities, such as likelihoods and prior probabilities) and the options in Fig. 10C allows for choosing parameters within each type.

Note, when the number of parameter samples exceeds the value in Fig. 9B, samples are thinned by a factor of two and subsequently gathered at half the rate (this is implemented to ensure that computational memory is not exhausted). The vertical dashed red line in Fig. 10A represents the so-called burn-in period (before which samples are discarded). To maximise efficiency this is dynamically shifted as more and more posterior samples are generated.

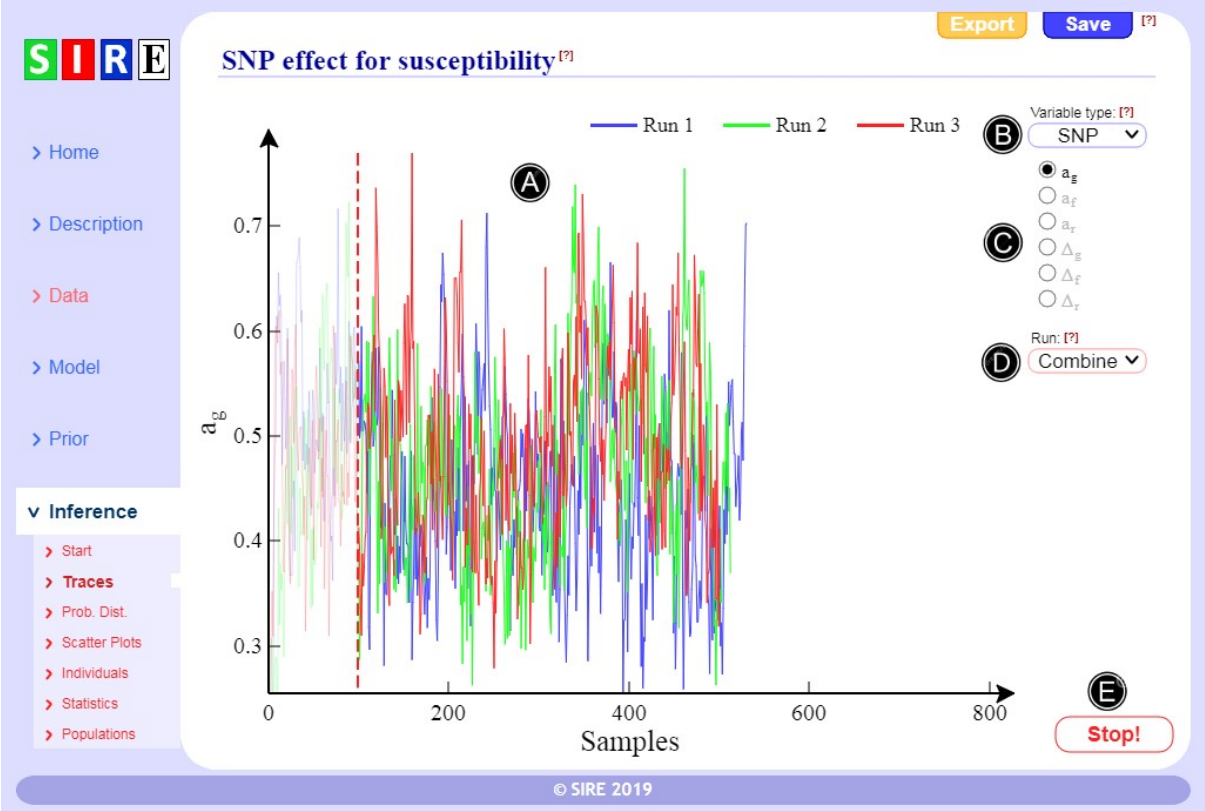


**Figure 10** – *Trace plot.* A: Posterior samples for selected parameter, B: variable type selection, C: variable selection, D: which runs should be displayed, E: stop gathering samples.

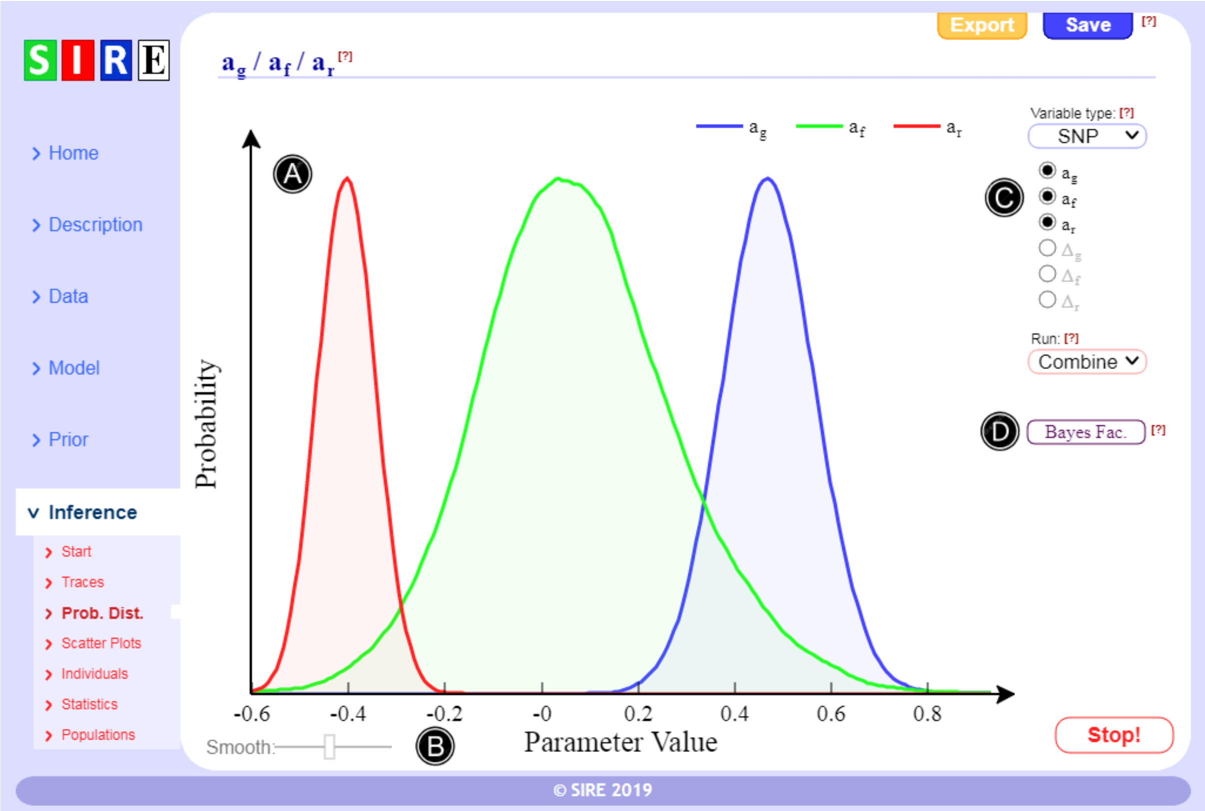


**Figure 11** – *Distributions.* A: Probability distribution(s) (use ctrl key to select multiple parameters), B: KDE smoothing parameter, C: parameter selection, D: calculate the Bayes' factor.

## 3.3 Probability distributions

The raw posterior samples from the previous section can be converted into posterior probability distributions, as shown in Fig. 11A. These are generated using a technique known as kernel density estimation (KDE) [3]. KDE makes use of a smoothing parameter which can be adjusted by means of the slider at Fig. 11B. This particular example simultaneously shows distributions for three model parameters, which can be achieved by holding down the control key and sequentially selecting the relevant parameters at Fig. 11C.

A Bayes factor (BF) is the ratio of the likelihood of one particular hypothesis to the likelihood of another [4]. The BF comparing the full model to one in which a particular parameter is fixed (usually to zero) can be calculated using the button at Fig. 11D. This is one way to determine statistically significant SNP and fixed effects affecting the three traits. A BF between 3 and 10 represent moderate evidence for one hypothesis over another and exceeding 10 is considered strong evidence.

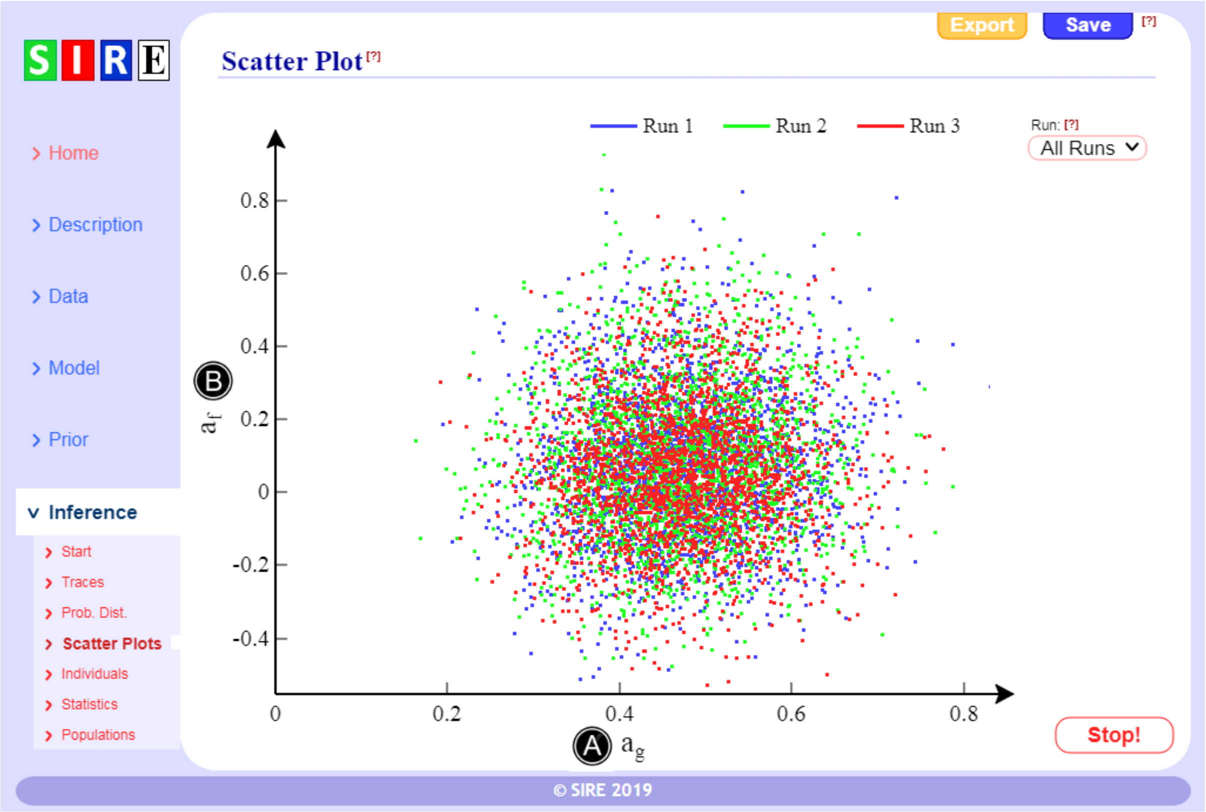


**Figure 12** – *Scatter plot.* A: Select $x$ variable, B: select $y$ variable.

## 3.4 Scatter plot

Scatter plots, as illustrated in Fig. 12, enable the user to display the posterior samples of one variable against another. This is achieved by means of clicking the $x$-axis (Fig. 12A) selecting the relevant variable and then doing the same for the $y$-axis (Fig. 12B). This particular example exhibits little correlation between the variables $a_g$ and $a_f$, but in other cases parameters can be highly correlated. Scatter plots are a useful tool to investigate confounding between different model parameters.

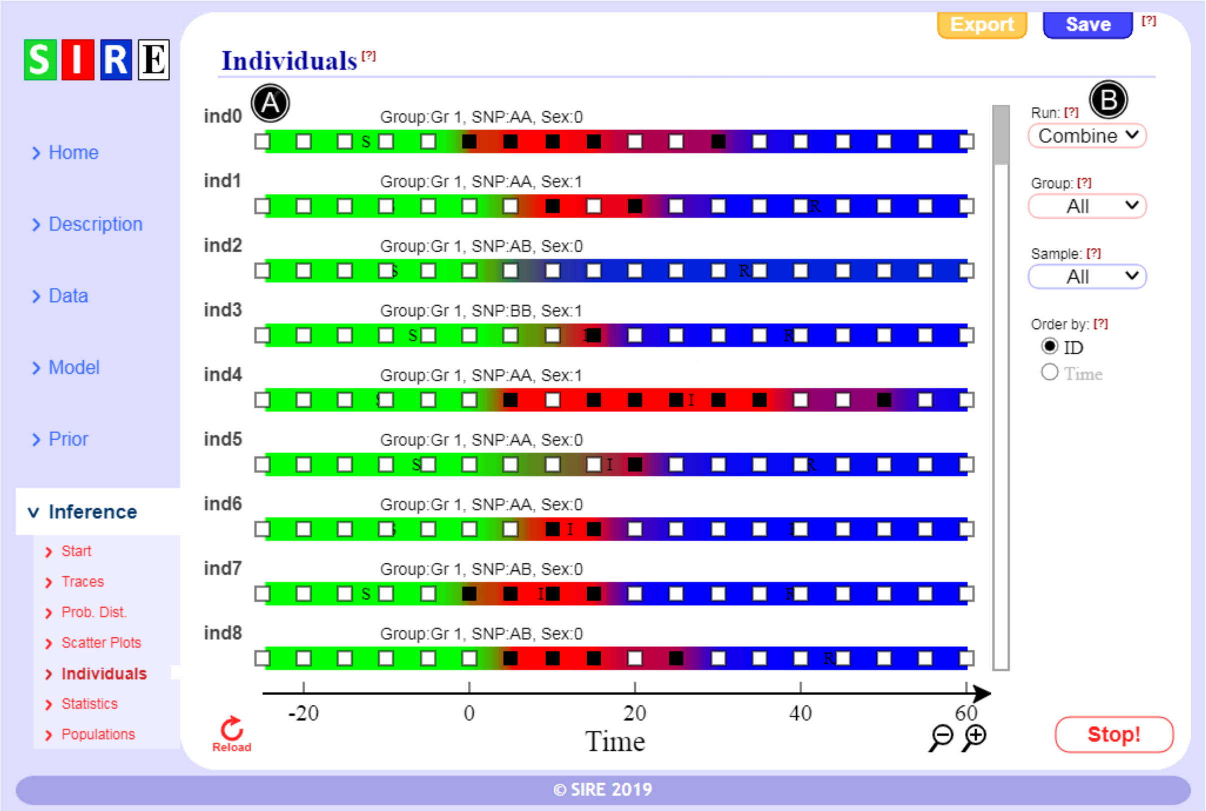


**Figure 13** –*Individual timeline plots.* A: Shows posterior distributions for individual timelines (taken from EX.7 which used disease diagnostic test results), B: various filters which can be applied.

## 3.5 Individual timelines

The view in Fig. 13A shows posterior distributions for individual timelines. Here the colours indicate the posterior probabilities for the disease status of individuals as a function of time. Green indicates that an individual is susceptible, red indicates infectious and blue indicates recovered. Gradations in colour between these extremes represent posterior uncertainty. Overlaid are the actual data (disease diagnostic test results in this particular example, where black/white squares indicate positive/negative outcome).

Various filters in Fig. 13B can be applied, such as only displaying individuals within a given contact group, a particular run or a given sample. Also the ordering can be based on individual ID or on the time at which they are first observed.

## 3.6 Statistics

SIRE summarises the posterior probability distributions (specifically the means and 95% credible intervals) for all the model parameters, as shown in Fig. 14. The credible intervals are of particular importance, because they can be used to establish if a particular SNP or fixed effect is statistically significant or not. For example, we note that the credible interval for $a_g$ in Fig. 14 goes from 0.137 to 0.428. This represents strong evidence that the genotype at the SNP truly does affect the susceptibility of individuals, because this range does not contain zero. On the other hand the same cannot be said for $a_f$, and so here nothing can definitively be concluded regarding SNP-based infectivity variation.

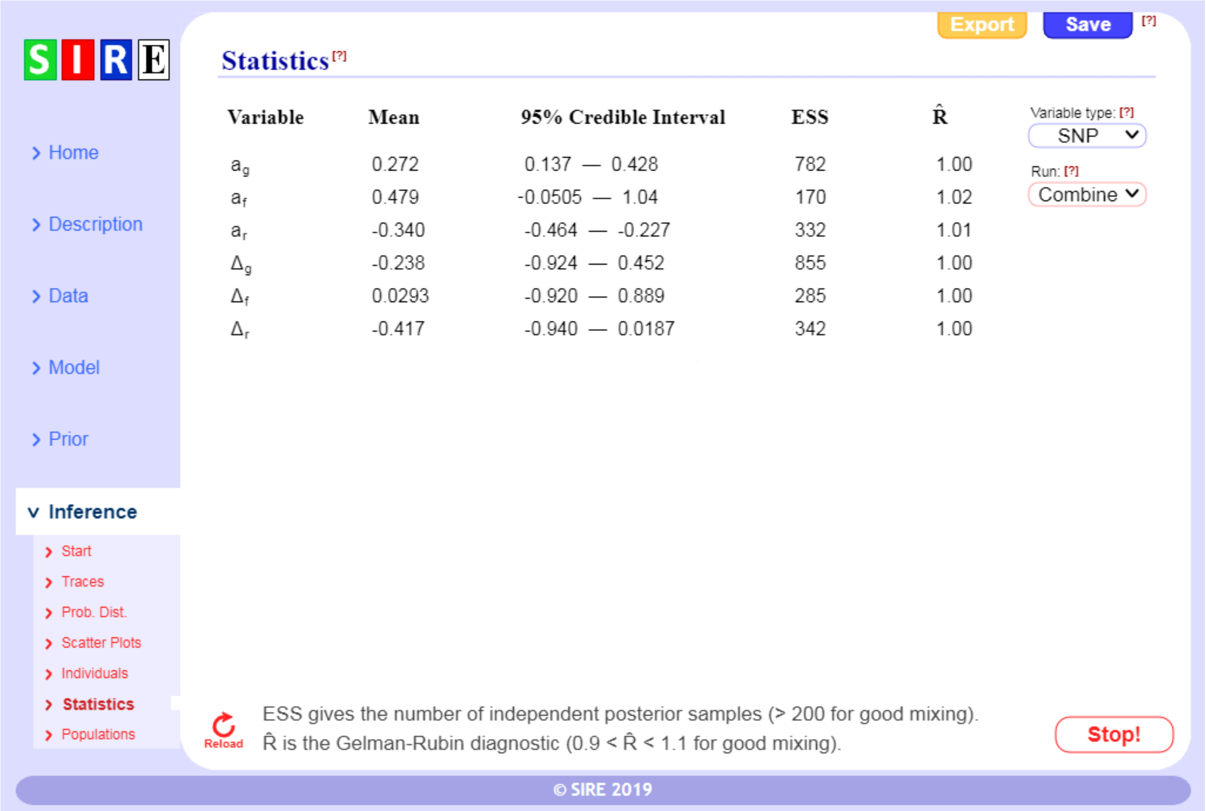


**Figure 14** –*Statistics.* A summary of posterior parameter estimates along with MCMC diagnostics.

Two measures are used to test for MCMC convergence:

- **Effective sample size (ESS)** – This estimates the number of truly independent posterior samples [2] (*i.e.* it accounts for correlations shown in the trace plots in section 3.2). Provided the ESS is greater than around 200 then posterior estimates can be reliably trusted. Because the ESS for $a_f$ in Fig. 14 is only 170 this indicates that SIRE needs to be run for longer to ensure reliable results. Note, the ESS is not always guaranteed to monotonically increase (in fact if it is less than 100 it often fluctuates wildly). However as its value increases these fluctuation should dampen as a result of the true posterior distribution being found.
- **The Gelman-Rubin statistic $\hat{R}$** – This checks that distributions from independent runs converge on the same posterior probability distribution [5]. Values between around 0.9 and 1.1 are considered to be indicative of convergence. If $\hat{R}$ fails to approach 1, even after a large number of iterations, this may be an indication of multimodality in the posterior distribution. This describes a scenario in which different MCMC chains become stuck in different local minima. This possibility can occur when there is only weak individual-based data, as is evident in [1] when SIRE was applied to final disease status data. Under these circumstances the results from SIRE cannot be trusted. Note this statistic relies on comparing independent MCMC runs, and so is not available when only a single run is being executed.

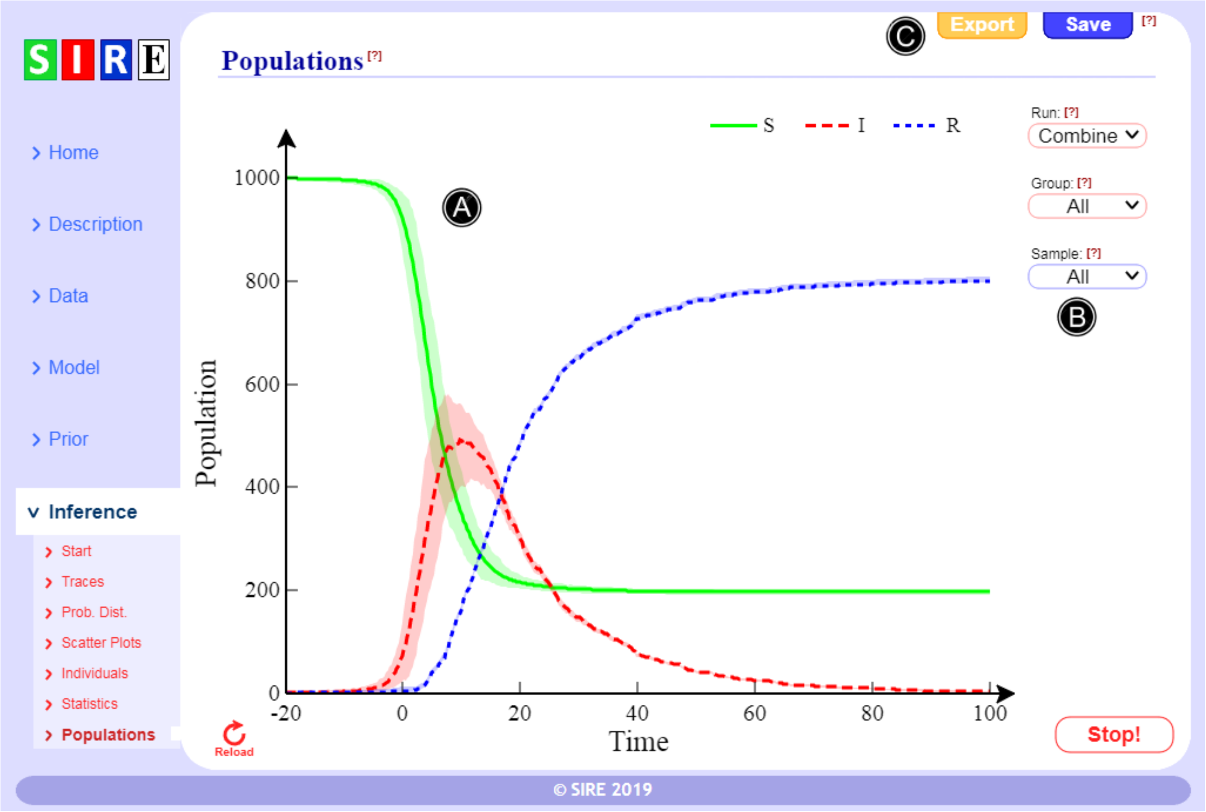


**Figure 15** –*Population plots.* A: The dynamic variation in the populations of susceptible, infected and recovered individuals (taken from EX.3), B: filters, C: exporting and saving.

## 3.7 Population plots

The number of susceptible, infected and recovered individuals can be plotted as a function of time, as illustrated in Fig. 15A. The lines represent posterior means and the shaded areas give 95% credible intervals. The results can be filtered by MCMC run, contact group or sample number (Fig. 15B).

## 3.8 Exporting

Exporting outputs can be achieved by clicking on the "Export" button on the top right hand corner (*e.g.* see Fig. 15C). A number of exporting possibilities exist:

- **Graph / Plot (.png)** – This outputs graphs (such as trace, scatter and population plots) as images. The output corresponds to the particular page being viewed.
- **Graph / Plot (.txt)** – This outputs the corresponding raw data as a text formatted table so they can be imported and plotted using other software.
- **Table (.txt)** – This outputs a text formatted table from the statistics page.
- **Parameters** – This outputs posterior parameter samples in text format (for analysis in other software). This consists of a table with sample number in the first column and a separate column for each of the model parameters. Note, this option can be selected on any of the inference pages.
- **Events** – This outputs posterior samples giving the raw infection and recovery times of individuals in text format (for subsequent analysis in other software). The first column of the table gives the sample number, the second gives the individual ID, and the third gives the life

history. Starting in the susceptible “S” state any transitions to the “I” and “R” states are recorded along with the times at which they occur.

## 3.9 Loading and saving

SIRE permits users to load and save analyses in a special “.sire” format (see Fig. 15C for saving and and Fig. 1B for loading). This is useful because it conveniently allows description, data and analysis to all be contained in a single file for future reference. Also those publishing results using SIRE can include the “.sire” file in the supplementary material such that analysis can transparently be reproduced by readers of the paper. When saving, two options are available: “With results” includes the posterior samples along with the model and data (so that inference does not need to be run again when the file is loaded), and “W/o results” which does not store the posterior samples (leading to a much smaller file size which can, for example, be emailed).

# 4 Examples

This section describes various examples chosen to illustrate potential applications of SIRE (see Fig. 1D).

For simplicity all examples assumed a scenario in which 1000 individual are split equally into 50 contact groups, each containing 20 individuals (an assessment of how parameter precisions vary as a function of these choices is given in [1]). Simulated data was generated by means of a modified Doob-Gillspie algorithm in which each contact group is assumed to be initially infected by a single infected individual. Data tables derived from these simulations are located in the subdirectory “Datasets” of the downloaded SIRE folder.

## 4.1 SIR model

Different disease scenarios (broadly these are classified as DS1: infection/recovery times known precisely, DS2: only recovery times known, DS3: only infection times known, DS4: periodic state data measurements and DS5: time censoring) are considered.

- **EX.1: Known infection and recovery times –** This example assumes that the infection and recovery times for all individuals are known (DS 1). Inspecting the posterior distributions for model parameters we find that overall they accurately represent the true parameter values used to simulate the data (see above). Data was taken from “Dataset 1.txt” in the “Datasets” folder.
- **EX.2: Staggered contact group timings –** Here the times at which different contact groups have epidemics are staggered. Again infection and recovery times for all individuals are assumed known (DS 1). Data was taken from “Dataset 2.txt”.
- **EX.3: Known recovery times** – This example assumes that only the recovery times for all individuals are known (DS 2). Data was taken from “Dataset 3.txt”.
- **EX.4: Disease transmission experiment** – This assumes that not only are the recovery times for all individuals known (DS 2), but also the initial disease status at t=0 is defined by the experiment. Data was taken from “Dataset 3.txt”.
- **EX.5: Known infection times** – This example assumes that only the infection times for all individuals are known (DS 3). Data was taken from “Dataset 3.txt”.

- **EX.6: Periodic disease status checks –** This example assumes that the disease status of individuals are measured periodically (DS 4). Data was taken from “Dataset 4.txt”.
- **EX.7: Disease diagnostic test results –** Here periodic disease diagnostic tests are performed on individuals (DS 4). The test is assumed to be sensitive to the *I* state. Data was taken from “Dataset 5.txt”.
- **EX.8: Disease diagnostic test results II –** Here two sets of diagnostic tests are made on individuals. The first is sensitive to the *I* state and the second is sensitive to both *I* and *R* states. Data was taken from “Dataset 5.txt”.
- **EX.9: Time censoring end of epidemics –** This illustrates data censoring (DS5), whereby the infection and recovery times for individuals are only observed up to a time before the end of epidemics. Data was taken from “Dataset 6.txt”.
- **EX.10: Time censoring beginning of epidemics –** This illustrates data censoring (DS5), whereby the infection and recovery times for individuals are only observed after epidemics have already started (*i.e.* missing the beginning). Data was taken from “Dataset 7.txt”.

## 4.2 SI model

This is a simplified model for diseases in which individuals do not recover/die. Again, different illustrative data scenarios are considered:

- **EX.11: Known infection and recovery times –** This example assumes that the infection and recovery times for all individuals are known (DS 1). Data was taken from “Dataset 8.txt”.
- **EX.12: Staggered contact group timings –** Here the times at which different contact groups have epidemics are staggered. Again infection and recovery times for all individuals are assumed known (DS 1). Data was taken from “Dataset 9.txt”.
- **EX.13: Periodic disease status checks –** This example assumes that the disease status of individuals are measured periodically (DS 4). Data was taken from “Dataset 10.txt”.
- **EX.14: Disease diagnostic test results –** Here periodic disease diagnostic tests are performed on individuals (DS 4). Data was taken from “Dataset 11.txt”.
- **EX.15: Time censoring end of epidemics –** This illustrates data censoring (DS5), whereby the infection and recovery times for individuals are only observed up to a time before the end of epidemics. Data was taken from “Dataset 12.txt”.
- **EX.16: Time censoring beginning of epidemics –** This illustrates data censoring (DS5), whereby the infection and recovery times for individuals are only observed after epidemics have already started (*i.e.* missing the beginning). Data was taken from “Dataset 13.txt”.

# 5 Code

The code for SIRE is split into two parts:

- **The interface** – This is written in javascript and runs on the desktop by means of NW.js. For those interested, the code consists of the “index.html” file in the main directory[6] and the javascript files in the “js” directory.

[6] On the Macintosh platform this is located in the “SIRE.app/Contents/Resources/app.nw/”folder.

- **The core code** – Performs the MCMC Bayesian analysis when SIRE is executed. This is written in highly efficient C++ code which can be found in the "Execute" directory (it consists of "sire.cc" along with numerous header files).

# 6 License and warranty

SIRE is free software under the terms of the GNU General Public License version 3 www.gnu.org/licenses/gpl-3.0.en.html. This allows users to redistribute and/or modify SIRE. The program is distributed in the hope that it will be useful, but WITHOUT ANY WARRANTY.

# 7 Citing SIRE

We kindly request that those who do use SIRE analysis in their publications cite this tool:

Pooley CM, Marion G, Bishop SC, Bailey RI, Doeschl-Wilson AB, *Estimating individuals' genetic and non-genetic effects underlying infectious disease transmission from temporal epidemic data*. bioRxiv. 2019:618363.

# 8 Plans for SIRE v2.0

Currently SIRE v1.0 is limited in a number of aspects. The next version of SIRE plans to incorporate the following improvements:

- Correlations in traits amongst related individuals (*i.e.* polygenic effects via genomic or pedigree based relationship matrices).
- Allow for missing contact group / SNP / covariate/ categorical data.
- Relax the closed contact group assumption to allow for individuals to enter and leave (or be culled) during epidemics.
- Extend the model to potentially include an exposed state (*i.e.* for individuals that are infected but not infectious).

# 9 Acknowledgments

SIRE makes use of two other pieces of software and we would like to acknowledge their contribution. Firstly, NW.js (from the website nwjs.io/) was used to build the interface. Secondly, tinyXML (from the website www.grinninglizard.com/tinyxml/) was used by the C++ code to parse the XML file which provides initialisation information. Both these software are excellent and highly recommended to others.

# References

1. Pooley CM, Bishop SC, Doeschl-Wilson AB, Marion G. Estimating genetic and non-genetic effects for host susceptibility, infectivity and recoverability using temporal epidemic data. bioRxiv. 2019:618363.
2. Geyer CJ. Practical Markov Chain Monte Carlo. Statist Sci. 1992;7(4):473-83.
3. Parzen E. On estimation of a probability density function and mode. The annals of mathematical statistics. 1962;33(3):1065-76.

4. Kass RE, Raftery AE. Bayes factors. J Am Stat Assoc. 1995;90(430):773-95.
5. Gelman A, Rubin DB. Inference from iterative simulation using multiple sequences. Stat Sci. 1992;7(4):457-72.
